# Ultrafast superconducting qubit readout with the quarton coupler

**DOI:** 10.1126/sciadv.ado9094

**Published:** 2024-10-09

**Authors:** Yufeng Ye, Jeremy B. Kline, Sean Chen, Alec Yen, Kevin P. O’Brien

**Affiliations:** ^1^ Department of Electrical Engineering and Computer Science, Massachusetts Institute of Technology, Cambridge, MA 02139, USA.; ^2^ Research Laboratory of Electronics, Massachusetts Institute of Technology, Cambridge, MA 02139, USA.

## Abstract

Fast, high-fidelity, and quantum nondemolition (QND) qubit readout is an essential element of quantum information processing. For superconducting qubits, state-of-the-art readout is based on a dispersive cross-Kerr coupling between a qubit and its readout resonator. The resulting readout can be high fidelity and QND, but readout times are currently limited to the order of 50 nanoseconds due to the dispersive cross-Kerr of magnitude 10 megahertz. Here, we present a readout scheme that uses the quarton coupler to facilitate a large (greater than 200 megahertz) cross-Kerr between a transmon qubit and its readout resonator. Full master equation simulations of the coupled system show a 5-nanosecond readout time with greater than 99% readout fidelity and greater than 99.9% QND fidelity. The quartonic readout circuit is experimentally feasible and preserves the coherence properties of the qubit. Our work reveals a path for order of magnitude improvements of superconducting qubit readout by engineering nonlinear light-matter couplings in parameter regimes unreachable by existing designs.

## Teaser

Proposed scheme using the quarton coupler is capable of an order of magnitude faster, 5-ns qubit readout with high fidelity.

## INTRODUCTION

Fast and high-fidelity qubit readout is essential for quantum error correction (*1*, *2*) and other feedback schemes in quantum computing and communication including teleportation (*3*, *4*) and state initialization (*5*, *6*). Superconducting qubits (*7*, *8*) are a leading material platform for quantum information processing (*9*) in part due to their reliably fast, high-fidelity, and quantum nondemolition (QND) readout (*10*–*12*). The state-of-the-art measurement scheme in superconducting circuits is dispersive readout (*10*, *12*), in which a linear coupling perturbatively gives rise to an effective nonlinear cross-Kerr interaction between a qubit and its auxiliary readout resonator. When the resonator is driven to a coherent state, this cross-Kerr interaction entangles the state of the qubit with the phase of the resonator coherent state. The coherent state then decays into the environment and is usually measured through heterodyne detection (*7*, *8*). Compared to other readout schemes such as high-power Jaynes-Cummings readout (*13*) or longitudinal readout (*14*), dispersive readout has experimentally demonstrated the fastest readout time (40 ns) (*12*), allowing high (>99%) readout fidelity (*11*, *12*) and high (>99%) QND fidelity (*12*, *15*).

High readout fidelity requires a high measurement signal-to-noise ratio (SNR) (*7*, *16*). The SNR for cross-Kerr–based qubit measurement over time *t* conveniently scales with (*7*, *16*)

$${\text{SNR}}^{2}\propto \eta \kappa \bar{n}{|\text{sin}\left(2\theta \right)|}^{2}t$$ (1)

where η is the quantum efficiency of the readout chain, κ is the decay rate of the resonator, and $\bar{n}$ is the average photon number in the resonator, meaning that $\eta \kappa \bar{n}$ is the effective rate at which measurement photons are collected. For a cross-Kerr of interaction strength χ, the term $|\text{sin}\left(2\theta \right)|=\frac{\chi \kappa }{\chi^{2}+\kappa^{2}/4}$ represents the amount of phase information each photon carries.

Over the past decades, there have been substantial advances in designing 2χ = κ to maximize ∣sin (2θ)∣ = 1 (*12*) and engineering devices including quantum-limited amplifiers that improve quantum efficiency η toward the theoretical maximum η = 1 (*17*–*19*). It has also become better understood that nonidealities in dispersive coupling tends to limit (*20*, *21*) the average readout resonator photon number $\bar{n}$ to low values $\bar{n}≲5$ (*11*, *22*) when the qubit is a state-of-the-art transmon (*23*). Notably, a simple way to improve the readout SNR is by increasing the coupling rate κ of the readout resonator to the environment. While a larger κ is easily achievable by increasing coupling capacitance, designs with κ/2π ≫ 10 MHz are practically difficult due to a number of reasons; many of which are ultimately caused by the perturbative nature of dispersive coupling.

Because κ ≈ 2χ is needed for optimizing the term ∣sin (2θ)∣, a larger χ is needed to accompany a larger κ; but dispersive χ for state-of-the-art transmon qubits (*23*) with low anharmonicity *E_C_*/2π ≈ 200 to 350 MHz are limited to χ/2π ≲ 10 MHz due to the perturbative cross-Kerr being limited to a small fraction of the qubit anharmonicity (*23*). In addition, the underlying linear coupling in dispersive coupling causes eigenstates of the qubit-resonator system to be combinations of qubit and readout resonator bare states, so a stronger resonator-environment coupling κ invariably increases many qubit eigenstate decoherence rates (*24*) such as Purcell decay (*25*). There is thus an opportunity to find a nonperturbative source of cross-Kerr interaction (*26*) between superconducting qubits and resonators that allows for a much larger χ and therefore a much larger κ, which can lead to a proportionally larger readout SNR.

Here, we show in simulation that, by designing high 2χ/2π ≈ κ/2π > 200 MHz, an order of magnitude higher than state-of-the-art dispersive readout, a cross-Kerr–based readout scheme can result in about an order of magnitude faster readout time—just 5 ns to reach 99% readout fidelity and 99.9% QND fidelity. To reach the high 2χ ≈ κ values, we leverage the quarton coupler we previously proposed in (*27*), which is capable of ultrastrong 2χ/2π → 1 GHz cross-Kerr coupling between a transmon qubit and resonator. This resonator is not the typical standing-wave waveguide mode but rather a linearized transmon with its intrinsic negative nonlinearity canceled by the quarton coupler’s induced positive nonlinearity. We will start by introducing the circuit for the proposed “quartonic readout” scheme. We then quantize the circuit and show in an example parameter study that the proposed quartonic readout scheme’s parameter requirements such as a large χ, good transmon resonator linearization, and sufficient transmon qubit anharmonicity can be satisfied. Last, we show full master equation simulations of the readout performance and discuss the preservation of qubit coherence properties as well as scalability of the proposed scheme.

## RESULTS

### Quartonic readout circuit

**Fig. 1.**
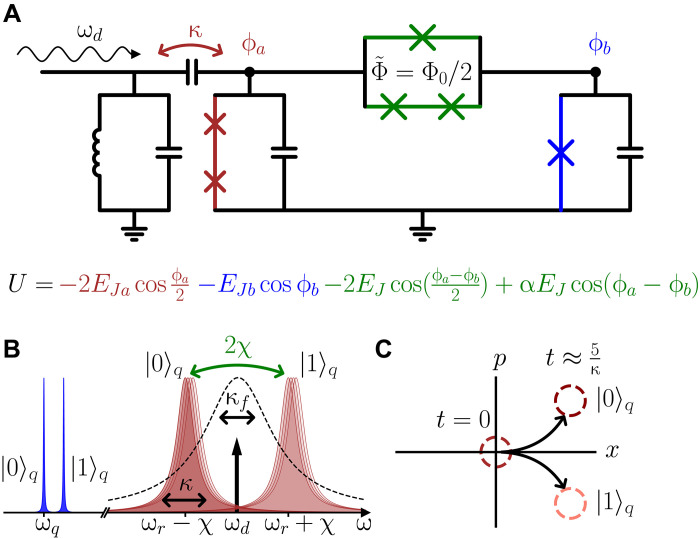
Circuit diagram and readout method. (**A**) The quarton (green) couples the linearized readout resonator (red) to the qubit (blue). A Purcell filter prevents qubit state leakage through the resonator. (**B**) Frequencies of the circuit. The readout drive at ω*_d_* = ω*_r_* is between the resonator frequencies for qubit state ∣0〉 and ∣1〉. The Purcell filter (dashed) is centered at ω*_r_*. (**C**) Qubit state–dependent evolution of the resonator coherent state.

We first describe the readout circuit, which uses the quarton, a purely nonlinear coupler introduced in our previous work (*27*). Using a quarton (green) to couple a low-anharmonicity transmon (red) and a transmon (blue), as depicted in Fig. 1A, yields a potential in terms of nodal superconducting phase ϕ

$$\begin{array}{cc} U= & -2E_{\mathrm{Ja}}\text{cos}\frac{\phi_{a}}{2}-E_{\mathrm{Jb}}\text{cos}\phi_{b}\\ & -2E_{J}\text{cos}\left(\frac{\phi_{a}-\phi_{b}}{2}\right)+\alpha E_{J}\text{cos}\left(\phi_{a}-\phi_{b}\right) \end{array}$$ (2)

Note that the quarton loop is biased by half a flux quantum (see Fig. 1A), leading to the positive sign of the α*E_J_* term (*27*). We can Taylor expand Eq. 2 to fourth order in ϕ to arrive at a more convenient form

$$\begin{array}{c} U\approx \frac{E_{\mathrm{Ja}}}{2}\frac{\phi_{a}^{2}}{2!}-\frac{E_{\mathrm{Ja}}}{8}\frac{\phi_{a}^{4}}{4!}+E_{\mathrm{Jb}}\frac{\phi_{b}^{2}}{2!}-E_{\mathrm{Jb}}\frac{\phi_{b}^{4}}{4!}\\ +E_{Q}\frac{{\left(\phi_{a}-\phi_{b}\right)}^{4}}{4!} \end{array}$$ (3)

where *E_Ja_*, *E_Jb_* are the Josephson energies of the Josephson junctions (JJs) in the two transmon-like modes, described by ϕ*_a_*, ϕ*_b_*; *E_J_* denotes the Josephson energy of each of the quarton’s series JJs, and $\alpha \approx \frac{1}{2}$ sets the Josephson energy of the quarton’s lone JJ to optimally cancel linear coupling. It is convenient to define *E_Q_* ≡ 3*E_J_*/8 to capture the effective quartic energy of the quarton potential.

We make three key observations about Eq. 3. First, the *E_Q_* term will supply a positive quartic $\left(+\phi_{i}^{4}\right)$ term, opposite to the $-\phi_{i}^{4}$ terms supplied by the *E_Ji_* terms. We can exploit this to linearize (keep only $\phi_{a}^{2}$) the ϕ*_a_* mode for use as a readout resonator while still allowing sufficiently large (now positive) self-Kerr of the ϕ*_b_* mode for use as a qubit. Second, the linear coupling, ϕ*_a_*ϕ*_b_*, from the quarton’s two branches (−2*E_J_* and α*E_J_* terms) cancel, leaving only nonlinear coupling terms, such as $\phi_{a}^{2}\phi_{b}^{2}$ between the qubit and resonator, which, when expanded in the Fock basis, contains the resonator-qubit cross-Kerr term *a*^†^*ab*^†^*b* needed for readout. Having cross-Kerr coupling without linear coupling prevents detrimental effects such as Purcell decay that are ubiquitous in state-of-the-art dispersive readout. Third, in contrast to the other nonlinear couplers (*28*, *29*), the quarton coupler does not supply any quadratic $\left(\phi_{i}^{2}\right)$ terms to directly change the linear Josephson inductance of the modes. In a linearized analysis (*30*), the quarton behaves as an electrical open circuit. This allows the cross-Kerr provided by the quarton coupler to scale approximately linearly with *E_Q_* (*27*), enabling ultrastrong cross-Kerr coupling 2χ/2π → 1 GHz.

We summarize some key operating frequencies of the quartonic readout system in Fig. 1B. The qubit state ∣0〉*_q_* → ∣1〉*_q_* and ∣1〉*_q_* → ∣2〉*_q_* transitions are detuned by a positive anharmonicity (from self-Kerr) and the readout resonator is nearly linear, with linewidth κ and cross-Kerr coupling 2χ to the qubit mode. Readout is performed by probing the resonator with a tone at ω*_d_*, between the two qubit state–dependent resonator frequencies. As in dispersive readout, this results in a coherent state evolution of the resonator mode as depicted in Fig. 1C, where, within a time *t* ≈ 5/κ, the qubit states are clearly distinguishable. Therefore, by using large κ ≈ 2χ, this enables high-fidelity readout with very short measurement time.

As shown in Fig. 1A, we also include a bandpass Purcell filter (*31*) of width κ*_f_* = 4κ (dashed line in Fig. 1B) to prevent qubit state leakage. Note that we must avoid flux biasing the galvanic loop formed by the quarton, the transmons, and ground while simultaneously allowing applied flux bias in the quarton loop $\tilde{\Phi }=\Phi_{0}/2$. This can be done using two flux bias lines, as in the asymmetrically threaded squid coupler (*32*).

### Optimal parameters for readout

Following Eq. 3 and adding the capacitive (kinetic) energy terms associated with Cooper pair number $\hat{n}$ operators, we can derive the total system Hamiltonian

$$\begin{array}{cc} \hat{H}= & 4E_{\mathrm{Ca}}{\hat{n}}_{a}^{2}+4E_{\mathrm{Cb}}{\hat{n}}_{b}^{2}+8E_{\mathrm{Cab}}{\hat{n}}_{a}{\hat{n}}_{b}-2E_{\mathrm{Ja}}\text{cos}\left(\frac{{\hat{\phi }}_{a}}{2}\right)-E_{\mathrm{Jb}}\text{cos}\left({\hat{\phi }}_{b}\right)\\ & +\alpha E_{J}\text{cos}\left({\hat{\phi }}_{a}-{\hat{\phi }}_{b}\right)-2E_{J}\text{cos}\left(\frac{{\hat{\phi }}_{a}-{\hat{\phi }}_{b}}{2}\right) \end{array}$$ (4)

Note that we have made the common approximation (*27*) to treat *n* series JJ arrays as a single element with potential *nE_J_* cos (ϕ/*n*) (see Supplementary Text for justification via a more detailed treatment without this approximation). We have also included a small capacitive coupling term $8E_{\mathrm{Cab}}{\hat{n}}_{a}{\hat{n}}_{b}$ to model the JJ capacitance in the quarton coupler and other stray capacitance between the resonator and qubit (assumed here to be ~5 fF). To maintain net zero linear coupling, we cancel the effect of the capacitive coupling term by engineering an opposite-signed linear inductive coupling in the quarton; this is done by perturbing the value of α ≈ 1/2 or “tilting” (*27*) the quarton (see Methods). We numerically solve (with ℏ=1 units) for the eigenenergies ω_*nanb*_ of Eq. 4 in the Fock basis, without any rotating wave approximations (RWAs), and label the eigenstates ∣*n_a_*, *n_b_*〉 by resonator (*n_a_*) and qubit (*n_b_*) excitation number (see Methods for details).

**Fig. 2.**
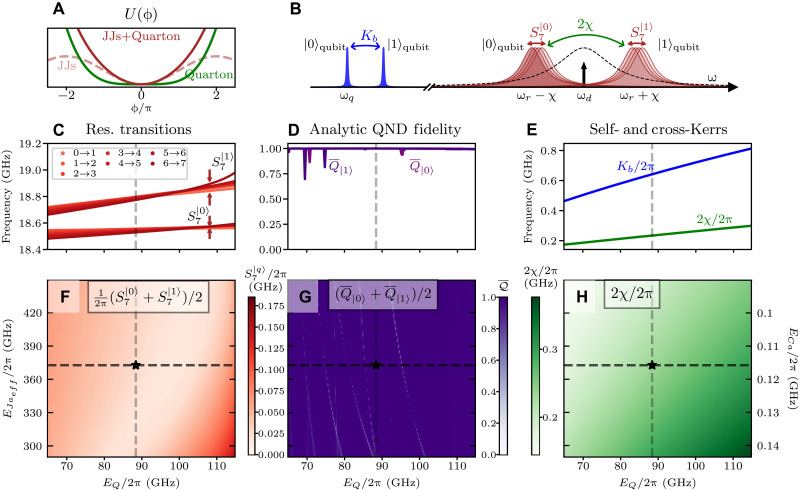
Parameter sweeps for optimal parameters for readout. (**A**) Linearizing the resonator by cancelling the JJ’s and quarton’s ϕ^4^ terms. (**B**) Schematic eigenenergy spectrum and labeled quantities to be optimized. (**C**) Resonator transition frequencies for qubit states ∣0〉, ∣1〉 showing good linearization at *E_Q_*/2π = 88.4 GHz. (**D**) Predicted QND fidelity from the analytic readout behavior as a function of *E_Q_*. (**E**) Cross-Kerr between the resonator and qubit (2χ) and qubit self-Kerr (*K_b_*) both increasing with *E_Q_*. (C) to (E) are the respective horizontal line cuts (black dashed line) in (F) to (H). (**F** to **H**) 2D sweeps of (F) frequency spread, (G) analytic QND fidelity, and (H) cross-Kerr, using constant resonator frequency $\omega_{a}=\sqrt{8E_{\mathrm{Ca}}E_{{\mathrm{Ja}}_{\mathrm{eff}}}}$, constant qubit parameters, and varying *E_Q_* and *E_Ja_eff__*/*E_Ca_*. The starred point has highly optimal parameters for readout.

For fast, high-fidelity, and QND qubit readout, the system should simultaneously exhibit low resonator nonlinearity, strong qubit-resonator cross-Kerr, strong qubit self-Kerr, and high predicted QNDness from analytics. We quantify the resonator nonlinearity by defining the resonator frequency spread $S_{n^{*}}^{|q\rangle }≔{\text{max}}_{i,j\leq n^{*}}|\left(\omega_{\mathrm{iq}}-\omega_{\left(i-1\right)q}\right)-\left(\omega_{\mathrm{jq}}-\omega_{\left(j-1\right)q}\right)|$, where n^*^ is the number of resonator transitions we consider, and (ω*_iq_* − ω_(*i*−1)*q*_) is the single-photon resonator transition from eigenstates ∣*i*, *q*〉 → ∣*i* − 1, *q*〉. We would ideally like our linearized transmon resonator to behave as a perfect linear resonator, or $S_{\infty }^{|q\rangle }=0$, but as shown in Fig. 2A, the linearized transmon resonator lives in an effective potential that is a sum of the transmon JJ’s cosine function and the quarton coupler’s first-order quartic function. This results in a quadratic function potential near the bottom of the potential well, with the corresponding low energy levels being linearly spaced but an increasingly less ideal quadratic potential and less linear energies for higher states. As such, we choose to linearize only the first n^*^ = 7 transitions and will drive the resonator to a low $\bar{n}=2$ coherent state in subsequent readout simulations to avoid exciting the higher, nonlinear resonator states. We also compute the resonator-qubit cross-Kerr 2χ = ω_11_ − ω_01 _− ω_10_ and qubit self-Kerr *K_b_* = ω_02_ − 2ω_01_, where ω*_ij_* is the energy of state *ij* in units of ℏ=1. The frequency spectrum of the qubit and resonator along with the metrics $S_{7}^{|q\rangle },2\chi ,K_{b}$ are schematically illustrated in Fig. 2B. To predict the QNDness of readout for qubit in state *k*, we use an analytic estimate, ${\bar{Q}}_{k}$, which assumes that the resonator quickly evolves into a steady-state coherent state (*22*) and then uses the Fermi’s Golden Rule to quantify leakage to noncomputational states caused by decay through the resonator (see Supplementary Text).

We now perform a parameter sweep with experimentally realistic parameters (see Table 1) to demonstrate how optimal parameters for readout can be found. We choose a transmon qubit with an uncoupled frequency of 5.7 GHz and a readout transmon resonator with an uncoupled frequency of ω*_a_*/2π = 18.5 GHz. We also use the previously defined *E_Q_* ≡ 3*E_J_*/8 from the quarton potential $U_{Q}\left(\phi \right)\approx \frac{E_{Q}}{4!}\phi^{4}+O\left(\phi^{6}\right)$ (*27*). In Fig. 2 (F to H), we sweep both *E_Q_* and the effective resonator Josephson energy *E_Ja_eff__* = *E_Ja_*/2 while keeping the resonator frequency approximately constant by fixing $\omega_{a}\approx \sqrt{8E_{\mathrm{Ca}}E_{{\mathrm{Ja}}_{\mathrm{eff}}}}$. At every point, we also solve for the optimal quarton tilt parameter 2α (which is heuristically optimized as described in Methods). The sweep reveals a trade-off between resonator linearization $\left(\text{minimizing}\;S_{7}^{|q\rangle }\right)$ and maximizing 2χ. In addition, we see that increasing *E_Ja_eff__* of the resonator increases the *E_Q_* that minimizes $S_{7}^{|q\rangle }$, consistent with Fig. 2A. See also Supplementary Text where we provide a more thorough analysis and summary of the Kerr effects in the system, including the general case of more than two series JJs and exact treatment of coupling terms such as (*b*^†2^ + *b*^2^)*a*^†^*a* (photon-enhanced squeezing). It is important to operate away from avoided crossings between eigenstates as they indicate strong hybridization of the resonator mode with the qubit mode, leading to qubit state leakage during readout and low QND fidelity (see dips in Fig. 2G).

**Table 1.** Summary of parameters for the starred point in Fig. 2.

Resonator frequency	ω_10_/2π	18.5 GHz
Resonator effective *E_J_*	*E_Ja_eff__*/2π = *E_Ja_*/2/2π	372.7 GHz
Resonator mode *E_C_*	*E_Ca_*/2π	115 MHz
Resonator capacitance	*C_a_*	169 fF
Qubit frequency	ω_01_/2π	6.55 GHz
Qubit *E_J_*	*E_Jb_*/2π	16.7 GHz
Qubit mode *E_C_*	*E_Cb_*/2π	267 MHz
Qubit capacitance	*C_b_*	72.5 fF
Quartic potential	*E_Q_*/2π	88.4 GHz
Quarton tilt	2α	1.02
Quarton lone, series JJ	α*E_J_*/2π, *E_J_*/2π	120.5 GHz, 235.7 GHz
Quarton junction capacitance	*C_J_*	5.20 fF
Resonator linearization	$S_{7}^{|0\rangle }/2\pi ,S_{7}^{|1\rangle }/2\pi$	28.3 MHz, 24.2 MHz
Qubit self-Kerr	*K_b_*/2π	642 MHz
Cross-Kerr	2χ/2π	234 MHz

In Fig. 2 (C to E), we take a horizontal line cut in the two-dimensional (2D) sweep (black dashed line in Fig. 2, F to H) to examine the effect of *E_Q_* alone. Figure 2C shows the resonator transition frequencies for both qubit states, and for various energy levels *i* → *i* + 1, a clear signature of linearization at *E_Q_*/2π = 88.4 GHz appears (gray dashed line), where the resonator frequency spread for both qubit states $S_{7}^{|0\rangle }$, $S_{7}^{|1\rangle }$ is less than 30 MHz. Furthermore, Fig. 2D illustrates the variations in the analytically predicted QND fidelity for both qubit states, showing that both ${\bar{Q}}_{|{}_{0,1}\rangle }>99.9\%$ at *E_Q_*/2π = 88.4 GHz. In Fig. 2E, we see the expected (*27*) trend of both the cross-Kerr and qubit self-Kerr *K_b_* increasing with increasing *E_Q_*. We note that this results in a positive self-Kerr for the qubit when *E_Q_* ≫ *E_Jb_*, a regime wherein the qubit’s potential landscape is dominated by the added $+E_{Q}\phi_{b}^{4}$ potential from the quarton coupler. At *E_Q_*/2π = 8.4 GHz, we find over 230 MHz of cross-Kerr and over 600 MHz of qubit self-Kerr. Because κ ≈ 2χ, this means that $S_{7}^{|q\rangle }\ll \kappa$ and we can drive the resonator to a coherent state. In summary, the point marked with a star in Fig. 2 (F to H) is an example of a parameter set (see Table 1) that satisfies all the criteria for optimal quartonic readout. We will proceed to use this parameter set for the subsequent readout dynamics simulations. We also emphasize that the proposed quartonic readout scheme is versatile and many other suitable parameter sets exists for other resonator and qubit frequencies.

### Readout performance

**Fig. 3.**
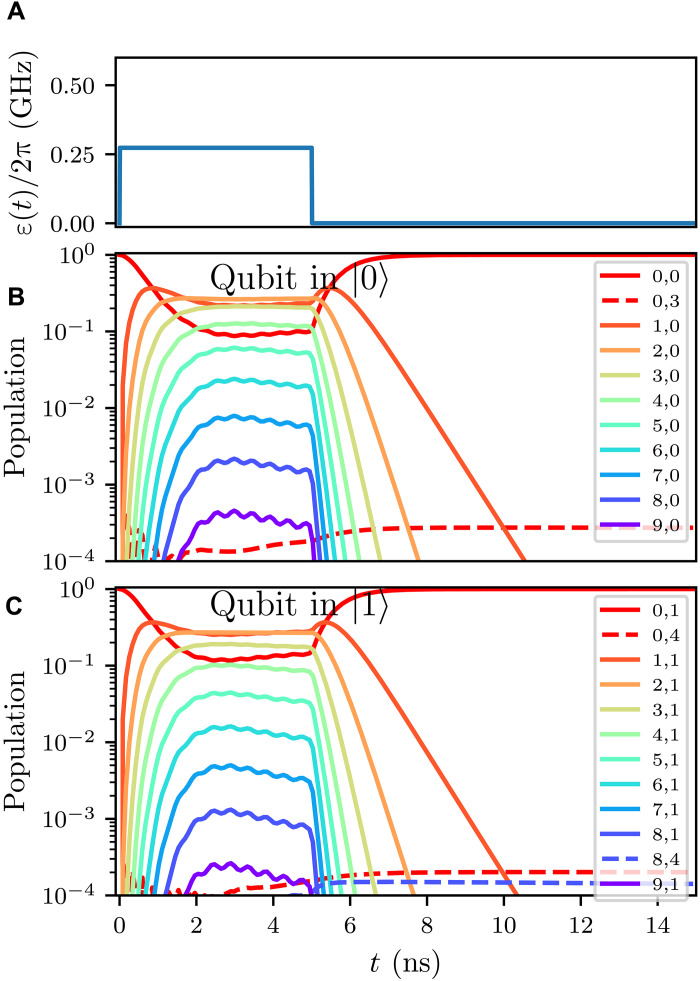
Quartonic readout simulation showing near-ideal evolution with high QNDness. (**A**) Square drive pulse for simulation. (**B** and **C**) Eigenstate population for readout of qubit initially in (B) qubit eigenstate ∣0〉 and (C) ∣1〉 with eigenstate convention ∣resonator, qubit〉. The state evolutions are highly QND with no substantial leakage into other qubit states.

**Fig. 4.**
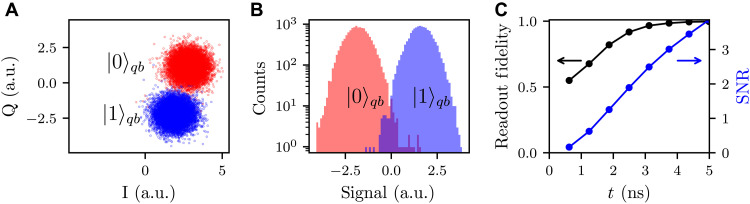
Quartonic readout statistics showing high readout fidelity for 5-ns square readout pulse. (**A**) Stochastic master equation simulated time-integrated measurement results in the in-phase (I) and quadrature (Q) plane; 12,800 points each represents a measurement trajectory, and red (blue) points are for qubit ∣0〉 (∣1〉). a.u., arbitrary units. (**B**) Histogram of the measurement signal from (A). (**C**) Readout fidelity and SNR over time (duration of square pulse).

Using the optimized system parameters in Table 1, we simulate performance with η = 1, κ/2π = 300 MHz and a readout drive resulting in steady-state $\bar{n}=2$ photons. Key performance results are summarized in Table 2. Numerical full master equation simulations show near-ideal state evolution over a readout pulse (Fig. 3A) duration of 5 ns with a high average QND fidelity of 99.96% in Fig. 3 and a high average readout fidelity of 99.72% in Fig. 4. We emphasize that we performed these simulations in the lab frame with the full JJ cosine potential and without applying RWAs and dispersive approximations (see Methods for more details).

**Table 2.** Summary of simulated transmon qubit readout performances.

Qubit initial state	Readout time (ns)	Readout fidelity	QND fidelity
∣0〉	5	99.71%	99.96%
∣1〉	5	99.73%	99.96%

As shown in Fig. 3A, we apply a resonator drive ε(*t*) = *u*(*t*)[2ε_0_ cos (ω*_d_t*)]*u*(5−*t*) for Heaviside step function *u*(*t*), which is a simple square pulse of width 5 ns followed by post-readout resonator ring-down time (10 ns). The resulting system dynamics are numerically computed with the Lindblad master equation solver in QuTiP (*33*) with all dissipations treated rigorously following ref. (*24*) (see Methods for details). In Fig. 3 (B and C), we plot the time evolution of the eigenstate (for the undriven Hamiltonian) population for qubit initialized in eigenstate ∣0〉 (Fig. 3B) and ∣1〉 (Fig. 3C), respectively. As expected, the large κ of quarton coupled readout resonator allows for very fast resonator response (≈1 ns) to the drive ε(*t*). We also observe a near-ideal behavior, with the resonator reaching an equilibrium coherent state from the drive while the qubit state remains mostly unchanged. Final states at the end of the readout and ring-down periods show very little leakage outside the starting qubit computational states, translating to a high QND fidelity of 99.96% (99.96%) for qubit initialized in ∣0〉 (∣1〉). Here, we use the standard definition of QND fidelity as being the probability of the qubit remaining in state ∣*i*〉 after measuring ∣*i*〉 (*15*). The excellent QND fidelity of our proposed readout results from the nonperturbative cross-Kerr between the readout resonator and qubit, and numerical simulation closely matches the analytic expectation of high $\bar{Q}$ in Fig. 2, justifying the construction of the analytic metric.

Simulated readout statistics are shown in Fig. 4. Here, we numerically compute measurement trajectories using the stochastic master equation (*34*) with heterodyne detection. We include all the same dissipations in the previous Lindblad master equation simulation, except we choose to monitor the dissipation from the relevant resonator decays for our measurement operator (see Methods). The 12,800 measurement trajectories are demodulated to extract the *I*(*t*), *Q*(*t*) quadratures, and following state-of-the-art experimental readout protocols (*11*), we simulate the integrated *I*, *Q* quadrature signals (see Fig. 4A) by time-integrating the trajectories *I*(*t*), *Q*(*t*) with weighting functions *W_Q_*(*t*) ∝ ∣〈*Q*_∣1〉_(*t*)−*Q*_∣0〉_(*t*)〉∣ and *W_I_*(*t*) ∝ ∣〈*I*_∣1〉_(*t*)−*I*_∣0〉_(*t*)〉∣. Each (*I*, *Q*) point in Fig. 4A is obtained by time-integrating a trajectory over the maximum readout pulse length of 5 ns, which, after an optimal axis projection, gives the histograms of signal in Fig. 4B. From Fig. 4B, a readout fidelity of 99.71%, 99.73% (for qubit initialized in ∣0〉, ∣1〉, respectively) can be extracted from histogram overlaps (*7*).

In Fig. 4C, we simulate the readout performance as a function of pulse length and plot the resulting readout fidelity and SNR. This is done by integrating the measurement trajectories over times ranging from 1 to 5 ns. Results here confirm that our proposed quartonic readout scheme with a high κ, χ readout resonator results in a much faster SNR and readout fidelity growth with measurement time. We note that measurement times beyond 5 ns could improve the readout fidelity further. Moreover, because the QND fidelity exceeds 99.9% for 5-ns readout, extending the readout to 50 ns would still achieve a QND fidelity above 99%.

## DISCUSSION

### Decoherence channels

The quarton coupler should not substantially affect the coherence time of the qubit. We analyze various decoherence channels and summarize the results in Table 3 (see Supplementary Text for details on each noise channel). We expect that the qubit will remain limited by dielectric loss, which is negligibly affected by the quarton coupler.

**Table 3.** Summary of quarton coupled qubit decoherence properties.

Mechanism	Decoherence rate Γ_1_	*T*_1_ = 1/Γ_1_
Purcell decay	$\kappa \left(\omega_{q}\right){|\langle 0,0|{\hat{n}}_{0}|0,1\rangle |}^{2}$	–
Quasiparticle	${\left|\langle 0|\text{sin}\frac{\hat{\phi }}{2}|1\rangle \right|}^{2}\frac{8E_{J}}{\pi \hbar }x_{\text{qp}}\sqrt{\frac{2\Delta }{\hbar \omega_{q}}}$	0.46 ms
Dielectric loss	$\frac{\hbar \omega_{q}^{2}}{8E_{C}Q_{\text{diel}}}{|\langle 0|\hat{\phi }|1\rangle |}^{2}\left[\text{coth}\left(\frac{\hbar \omega_{q}}{2k_{B}T}\right)+1\right]$	0.285 ms
Flux noise	${\left|\langle 0|\frac{\partial \hat{H}}{\partial \Phi }|1\rangle \right|}^{2}S_{\Phi }\left(\omega_{q}\right)$	2.1 ms
	**Dephasing rate** **Γ_2_**	***T*_2_ = 1/Γ_2_**
Thermal photon	$\frac{{\bar{n}}_{t}\left({\bar{n}}_{t}+1\right){\left(2\chi \right)}^{2}}{\kappa }$	5.86 ms
Flux noise	Simulated	11.4 ms

### Future directions

The high fidelities simulated using only a simple square pulse demonstrate the robustness of our proposed scheme and leave ample room for further improvements. For instance, existing dispersive readout optimization techniques such as pulse shaping (*35*, *36*) or qubit shelving (*37*) should be readily applicable to our quartonic readout scheme for some constant factor improvements in measurement time. Another avenue of improvement could be to leverage the inherent nonlinearity in our transmon-based readout resonator for bifurcation (*12*) to enhance readout performance. More comprehensive parameter sweep and optimization could also show operation points with small $S_{n^{*}}^{|q\rangle }$ for a larger n^*^, which allows for more photons $\bar{n}$ to be used in readout for an even higher SNR. However, a higher $\bar{n}$ is also known to cause deleterious effects such as reduced qubit *T*_1_ (*38*) and qubit ionization (*39*) or measurement-induced state transitions (*20*). We show in the Supplementary Materials that our current operating point has $\bar{n}$ far below the critical readout photon number for ionization.

We also emphasize that, while our results here use the much higher available SNR of quartonic readout to reduce measurement time, it is conceivable that important use cases such as large-scale error correction setups (*1*) could prioritize hardware efficiency over fast feedback time. It may therefore be advantageous to instead use the higher available SNR on tolerating a lower measurement quantum efficiency η in the hardware setup, thereby removing the need for quantum-limited amplifiers (*17*–*19*) and their accompanying impedance-matching circulators or isolators (*18*, *19*). Because these quantum-limited amplifiers typically improve η by about 10 times (*8*) and quartonic readout improves the readout SNR by more than 10 times, we envision that state-of-the-art 50-ns or less measurement time (*37*) without quantum-limited amplifiers should be feasible, representing a drastic reduction in measurement hardware complexity.

### Feasibility and scalability

All proposed superconducting and normal metal circuit necessary for quartonic readout should be compatible with standard microfabrication. See Supplementary Text for more detailed studies on robustness to fabrication variances in *E_J_* and deviations in flux bias $\tilde{\Phi }$.

For important use cases such as large-scale error correction setups (*1*), it is necessary to have frequency-multiplexed readout for hardware efficiency. This seems difficult at first glance as resonators typically must be spaced several linewidths apart to avoid cross-talk (*40*, *41*) which, for large κ/2π = 300 MHz, can strain the few gigahertz bandwidth of analog electronics (*42*). However, it has been shown in (*41*) that the necessary frequency spacing between each resonator can be greatly reduced with individual Purcell filters, so this may provide a path to engineering multiplexed ultrafast quartonic readout of many qubits in the future. Alternatively, by sacrificing some readout speed, κ/2π = 100 to 200 MHz designs can easily accommodate 10 multiplexed qubit readout channels with existing hardware (*42*) while still operating in a parameter regime that is difficult or impossible to reach for dispersive readouts with 2χ ≈ κ ≲ 2π × 10 MHz. Frequency multiplexing would also be much easier if one opted to use the extra SNR from quartonic readout toward lowering η rather than lowering the measurement time as this makes measurement pulses much longer in time and thus narrower in frequency, thereby reducing cross-talk.

Microwave measurement hardware compatible with higher-frequency measurements have also been developed (*43*). Because higher frequencies are typically less used, the ability to operate readout resonators at a much higher frequency due to the quarton coupler’s nonperturbative cross-Kerr not being explicitly frequency dependent may be an important practical advantage compared to state-of-the-art dispersive readout, which suffers from frequency crowding (*40*).

### Takeaways

In summary, we present an experimentally feasible, quarton coupler–based scheme for ultrafast superconducting qubit readout. Simulation results show that only 5 ns is needed to reach a readout fidelity above 99% and QND fidelity above 99.9%, implying order of magnitude potential improvements to state-of-the-art experiments that require 40 ns (*12*) and could thus lead to much faster feedback schemes such as quantum error correction protocols (*1*, *2*). In addition to the immediate benefits in readout speed, the ability of the quartonic readout scheme to operate with a low readout photon number ($\bar{n}=2$) and huge qubit-resonator detuning could alleviate some practical readout-related issues such as chaos (*21*), thermal photon dephasing (*44*), and frequency crowding (*40*). Unlike many existing directions of superconducting qubit readout improvements that focus on optimizing fundamentally constrained parameters like the quantum efficiency η ≤ 1 (*17*–*19*) and dispersive cross-Kerr and environmental coupling 2χ ≈ κ ≲ 10 MHz (*11*, *12*, *15*), our work suggests that better nonperturbative nonlinear couplers can overcome traditional design constraints to reach previously unexplored regimes of much larger χ, κ and thus greatly improved readout speed.

## METHODS

### Fock basis treatments

With a nonperturbative coupler between qubit and resonator modes, the circuit’s eigenstates may differ substantially from a naive set of basis states. We represent the circuit eigenstates with the Fock basis, i.e., the Hilbert space is spanned by a tensor product of Fock states in the resonator and qubit mode subspace. Using the Fock basis is convenient because its basis states can be chosen to be very close to the eigenstates. This is essential for using heuristics such as minimizing linear coupling terms (*a*^†^*b*) but maintaining the cross-Kerr terms *a*^†^*ab*^†^*b*.

The first step is writing the Hamiltonian of Eq. 4 in some abstract Fock basis with the canonical transformations ${\hat{\phi }}_{j}\to \phi_{{\mathrm{zpf}}_{j}}\left(a_{j}^{†}+a_{j}\right)$, ${\hat{n}}_{j}\to {\text{in}}_{{\mathrm{zpf}}_{j}}\left(a_{j}^{†}-a_{j}\right)$ and then normal ordering and separating out all the coupling terms to obtain a Hamiltonian of the form

$$H=H_{a}+H_{b}+H_{\text{coup}}$$ (5)

where each *H_j_* ∈ {*H_a_*, *H_b_*} has all terms that only contain the operators $a_{j},a_{j}^{†}$, and each term in *H_coup_* contains operators from both modes. Normal ordering is important as this allows the process to be analytic and the coupling term *H_coup_* to be as simplified as possible.

To select an accurate basis to represent each mode *j*, we need to optimize our numerical choices of $\phi_{{\mathrm{zpf}}_{j}}=\frac{1}{2n_{{\mathrm{zpf}}_{j}}}$. We do this by having the Fock basis states of mode *j*, {∣0*_j_*〉, ∣1*_j_*〉, … ∣*N_j_*〉}, to be as close as possible to the eigenstates of *H_j_*, $\left\{\left|e_{0}^{\left(j\right)}\rangle ,\ldots \right|e_{N}^{\left(j\right)}\rangle \right\}$. Specifically, we aim to maximize the quantity

$$\sum_{k=0}^{N_{j}}{\left|\langle k_{j}|e_{k}^{\left(j\right)}\rangle \right|}^{2}$$

where we choose *N_j_* depending on the number of energy transitions we are interested in, which is usually up to 10. Because each bare Hamiltonian *H_j_* includes terms with ϕ*_zpf__j_* from the other mode, the optimization of ϕ*_zpf__j_* for each basis is not completely independent. However, using the procedures and heuristics explained in Supplementary Text, we are able to consistently achieve overlap probabilities of over 98% between the Fock basis states and the eigenstates of *H_j_* for the first 10 energy levels in each mode. This is usually sufficient for labeling the relevant eigenenergies and our relevant heuristics.

We then label the eigenstates of *H* by iterating through each of the Fock basis states ∣*n_a_*, *n_b_*〉 = ∣*n_a_*〉 ⊗ ∣*n_b_*〉 and identifying the eigenstate ∣λ〉 (of *H*) with greatest overlap ∣〈*n_a_*, *n_b_* ∣ λ〉∣^2^. Then, ∣λ〉 is labeled as the eigenstate version of ∣*n_a_*, *n_b_*〉. When the terms in *H_coup_* are not causing near degeneracies of *H_j_* eigenstates, the labeling choices are clear due to the high (over 98%) overlap of *H_j_* eigenstates with the basis states. As described in the main text, degeneracies between the resonator and qubit excitations can lead to higher leakage rates and lower QND fidelity, so we aim to ensure high overlaps between *H_j_* (bare) eigenstates and *H* (dressed) eigenstates.

### Quarton tilting

To minimize Purcell decay and unwanted mixing of the qubit and resonator modes, a main function of the quarton coupler is to have strong nonlinear (cross-Kerr) coupling without linear coupling. However, even a purely quartic ${\left({\hat{\phi }}_{a}-{\hat{\phi }}_{b}\right)}^{4}$ coupling term, when expressed in the Fock basis with normal ordering, introduces some weak Jaynes-Cummings *a*^†^*b* + *ab*^†^ coupling terms, exactly analogous to a linear coupling term ${\hat{\phi }}_{a}{\hat{\phi }}_{b}$. This, along with unavoidable linear capacitive coupling terms ${\hat{n}}_{a}{\hat{n}}_{b}$, motivates us to modify the quarton potential by adjusting the tilt *t* ≔ 2α to minimize the linear coupling (*27*). With a Fock basis close to the bare (*H_j_* in Eq. 5) eigenstates, we can tilt the quarton to minimize the quantity

$${\left|\left(\langle 0_{a}|⊗\langle 1_{b}|)H(|1_{a}\rangle ⊗|0_{b}\rangle \right)\right|}^{2}$$

where *H* is the full circuit Hamiltonian and ∣*n_j_*〉 is the *n*-photon Fock state in mode *j*. This can be done by sweeping values of *t*, where we reoptimize the basis as described previously and calculate the linear coupling for each *t*.

### Master equation simulation

By choosing the Lindblad master equation formalism, we implicitly make the standard approximations (*45*) in its derivation (e.g., Markovian and Born). We discuss the validity of this in Supplementary Text on approximations in the master equation. We follow (*24*) in constructing the Lindblad dissipators in the master equation by assuming a zero-temperature bath and allowing only high-to-low energy eigenstate transitions; it is also necessary to group transitions close (≤κ) in frequency to the same dissipator as these transitions have correlated coupling to the same bath mode. In stochastic master equation simulations, the same Lindblad dissipators are used and the monitored stochastic operator is set to include only the resonator transitions for the first few qubit states. For computational efficiency, when simulating the quartonic readout system, we truncate the otherwise huge Fock basis Hilbert space and keep only the low photon number subspaces with a threshold determined iteratively via convergence of results. For stochastic master equation simulations, 12,800 trajectories are used with 200 simulation substeps for a measurement generating time step of 1/(5ω*_d_*). See Supplementary Text on master equation simulations for a more detailed presentation.

## Supplementary Materials

### This PDF file includes:

Supplementary Text Figs. S1 to S7 Tables S1 and S2 References
