# Supplementary material for: Ultrafast superconducting qubit readout with the quarton coupler
Source: Sci Adv. 2024 Oct 9;10(41):eado9094. doi: 10.1126/sciadv.ado9094 (PMC11642098; doi:10.1126/sciadv.ado9094)
Supplement: Supplementary file 1 — Supplementary Text Figs. S1 to S7 Tables S1 and S2 References [file sciadv.ado9094_sm.pdf]

Science Advances
AAAS

Supplementary Materials for

Ultrafast superconducting qubit readout with the quarton coupler

Yufeng Ye *et al.*

Corresponding author: Kevin P. O'Brien, kpobrien@mit.edu

Sci. Adv. **10**, eado9094 (2024)
DOI: 10.1126/sciadv.ado9094

This PDF file includes:

Supplementary Text
Figs. S1 to S7
Tables S1 and S2
References

Analytic QND fidelity estimate

Here we provide a detailed construction of the analytic QND fidelity estimate used in the main text.

We begin by approximating the system steady-state during readout as $|\alpha, k\rangle$ where the resonator is in a coherent state $|\alpha\rangle$ and the qubit is in its original number state $|k\rangle$. This is valid both in dispersive readout (*22*) and in our quartonic readout for sufficiently good readout resonator linearization for the given drive and κ, as demonstrated in the main text. We treat qubit leakage as a small perturbation to this steady state, caused by some weak decay following Fermi's Golden Rule that takes $|\alpha, k\rangle \rightarrow |n, q\rangle$ where $|n, q\rangle$ is some eigenstate with a different qubit state $q \neq k$. We focus only on the leakage caused by incoherent decay and neglect leakage caused by coherent drive because the leakage transitions are typically far off-resonant from our readout drive frequency, so the coherent drive induced leakage is negligible compared to incoherent decay. It is convenient now to introduce a decay-induced transition matrix:

$$\begin{aligned} D &= \sum_{i<j} |e_i\rangle \langle e_j| \times \sqrt{\kappa_{\text{eff},ij}} \\ &= \sum_{i<j} |e_i\rangle \langle e_j| \times |\langle e_i| \hat{n}_0 |e_j\rangle| \sqrt{\kappa(\omega_{ij}) f(\omega_{ij})} \end{aligned} \tag{S1}$$

which maps any energy eigenstate $|e_j\rangle$ to lower energy eigenstates $|e_i\rangle$ (here i, j are indices sorted by low to high energy) with an effective rate $\kappa_{\text{eff},ij}$. The effective rate is weighted by the transition's normalized charge ($\hat{n}_0$) coupling to the bath, the bath density of state at the transition's frequency ω_{ij}, and (optionally) the filtering function $f(\omega)$ of the Purcell filter. This makes the usual assumption that the bath is at zero temperature and so the system can only lose energy to the bath. We also use our eigenstate labelling result to assign each eigenstate to some unique resonator, qubit state $|e\rangle = |n, q\rangle$.

Using the transition matrix D and ignoring second-order processes like reverse leakage back to original qubit state $|q \neq k\rangle \rightarrow |k\rangle$, we can estimate the first-order leakage rate from the readout steady state $|\alpha, k\rangle$ as:

$$\Gamma_{|k\rangle} = \sum_{n, q \neq k} |\langle n, q| D |\alpha, k\rangle|^2 \tag{S2}$$

which estimates the leakage rate as the sum of decay rates to eigenstates that do not preserve qubit state ($q \neq k$). Finally, for readout times Δt and ignoring transient response, we can estimate the analytic QND fidelity ($\bar{\mathcal{Q}}$) from leakage:

$$\begin{aligned} \bar{\mathcal{Q}}_{|k\rangle} &= \exp\left(-\Delta t \times \Gamma_{|k\rangle}\right) \\ &= \exp\left(-\Delta t \sum_{n, q \neq k} |\langle n, q| D |\alpha, k\rangle|^2\right) \end{aligned} \tag{S3}$$

In summary, $\bar{\mathcal{Q}}_{|k\rangle}$ is an analytic estimate of the readout QND fidelity ($\mathcal{Q}_{|k\rangle}$) that accounts for leakage of population into different qubit number eigenstates over the course of a short readout time Δt by an ideal readout state $|\alpha, k\rangle$.

Full circuit Hamiltonian (without series JJ approximation)

Here we show that it can be safe to ignore the internal modes (also known as "collective modes" in fluxonium literature (*46*)) of the series JJ chains in the circuit. As shown in Fig. S1, for the representative quartonic readout circuit proposed in the main text, there are two internal modes associated with the free nodes ϕ_q, ϕ_r in the quarton and readout resonator, respectively.

The potential energy of the circuit can be exactly expressed in terms of the nodes $(\phi_a, \phi_b, \phi_q, \phi_r)$, assuming half flux quantum bias in the quarton loop:

$$\begin{aligned} U = &-E_{Ja}[\cos(\phi_a - \phi_r) + \cos(\phi_r)] - E_{Jb}\cos\phi_b \\ &- E_J[-\alpha\cos(\phi_a - \phi_b) + \cos(\phi_a - \phi_q) + \cos(\phi_q - \phi_b)] \end{aligned} \tag{S4}$$

Using trig identities, we can re-write this as:

$$\begin{aligned} U = &-2E_{Ja}\cos\frac{\phi_a}{2}\cos\frac{\phi_a - 2\phi_r}{2} - E_{Jb}\cos\phi_b \\ &- E_J[-\alpha\cos(\phi_a - \phi_b) + 2\cos(\frac{\phi_a - \phi_b}{2})\cos(\frac{\phi_a - 2\phi_q + \phi_b}{2})] \end{aligned} \tag{S5}$$

which is very close to the simplified form we used in the main text:

$$\begin{aligned} U_{\text{eff}} = &-2E_{Ja}\cos\frac{\phi_a}{2} - E_{Jb}\cos\phi_b \\ &- E_J[-\alpha\cos(\phi_a - \phi_b) + 2\cos(\frac{\phi_a - \phi_b}{2})] \end{aligned} \tag{S6}$$

This motivates us to define a change of variable,

$$\begin{aligned} \tilde{\phi}_r &= \frac{\phi_a - 2\phi_r}{2} \\ \tilde{\phi}_q &= \frac{\phi_a - 2\phi_q + \phi_b}{2} \end{aligned} \tag{S7}$$

which together with the unchanged ϕ_a, ϕ_b defines the transformation:

$$\begin{aligned} &\vec{\phi}' = W\vec{\phi} \\ &\begin{bmatrix} \phi_a \\ \phi_b \\ \tilde{\phi}_r \\ \tilde{\phi}_q \end{bmatrix} = \begin{bmatrix} 1 & 0 & 0 & 0 \\ 0 & 1 & 0 & 0 \\ 1/2 & 0 & -1 & 0 \\ 1/2 & 1/2 & 0 & -1 \end{bmatrix} \begin{bmatrix} \phi_a \\ \phi_b \\ \phi_r \\ \phi_q \end{bmatrix} \end{aligned} \tag{S8}$$

This transformation W is not unitary, so in order to maintain the canonical commutation relations between all superconducting phase $\hat{\phi}$ and Cooper pair number $\hat{n}$ operators:

$$[\phi_i, \phi_j] = 0, [n_i, n_j] = 0, [\phi_i, n_j] = i\delta_{ij} \tag{S9}$$

we must also transform the $\vec{n}$ by (*47*):

$$\begin{gathered}\vec{n'} = (W^\top)^{-1}\vec{n} \\ \begin{bmatrix} \tilde{n}_a \\ \tilde{n}_b \\ \tilde{n}_r \\ \tilde{n}_q \end{bmatrix} = \begin{bmatrix} 1 & 0 & 1/2 & 1/2 \\ 0 & 1 & 0 & 1/2 \\ 0 & 0 & -1 & 0 \\ 0 & 0 & 0 & -1 \end{bmatrix} \begin{bmatrix} n_a \\ n_b \\ n_r \\ n_q \end{bmatrix}\end{gathered} \tag{S10}$$

This transforms the capacitive energy of the circuit via:

$$\begin{aligned} T &= \frac{4e^2}{2}\vec{n}^\top C^{-1}\vec{n} \\ &= \frac{4e^2}{2}\vec{n}'^\top W C^{-1} W^\top \vec{n}' \\ &= 4\vec{n}'^\top \left(\frac{e^2}{2} W C^{-1} W^\top\right)\vec{n}' \\ &= 4\vec{n}'^\top \overset{\leftrightarrow}{E_C}\, \vec{n}' \end{aligned} \tag{S11}$$

where the capacitive energy matrix $\overset{\leftrightarrow}{E_C}$ is approximately diagonal but has small off-diagonal terms arising from finite junction capacitance of JJs. For instance, with experimentally-realistic estimates of capacitances: $\{C_{J,q}, C_{J,r}, C_a, C_b\} = \{3, 7.5, 80, 70\}$ fF:

$$\overset{\leftrightarrow}{E_C} = \begin{pmatrix} 223.6 & 9.2 & 0 & 0 \\ 9.2 & 265.7 & 0 & 0 \\ 0 & 0 & 1291.3 & 0 \\ 0 & 0 & 0 & 3228.4 \end{pmatrix} \text{MHz} \tag{S12}$$

with energy in units of $h = 1$. This makes intuitive sense, as the junction capacitances in the quarton creates a direct (for α JJ) and indirect (for series JJ) path for capacitive coupling (non-zero $E_{C,12}$) between the two transmons; but the junction capacitances in the resonator do not contribute to any coupling.

Putting everything together, the total circuit Hamiltonian with transformed variables is:

$$\begin{aligned} H &= U + T \\ &= -2E_{Ja}\cos\frac{\phi_a}{2}\cos\tilde{\phi}_r - E_{Jb}\cos\phi_b - E_J[-\alpha\cos(\phi_a - \phi_b) + 2\cos(\frac{\phi_a - \phi_b}{2})\cos\tilde{\phi}_q] \\ &+ 4E_{C,11}\tilde{n}_a^2 + 4E_{C,22}\tilde{n}_b^2 + 8E_{C,12}\tilde{n}_a\tilde{n}_b + 4E_{C,33}\tilde{n}_r^2 + 4E_{C,44}\tilde{n}_q^2 \end{aligned} \tag{S13}$$

It is clear from Eq. (S13) that the quarton and resonator internal modes are transmon-like, with cosine potential $-2E_{J(a)}\cos\phi_{q(r)}$, capacitive energy $4E_{C,44(33)}\tilde{n}^2_{q(r)}$, and no capacitive coupling. Their only coupling to the resonator, qubit modes a, b is through nonlinear terms

$\cos{(\phi_{a,b})}\cos(\phi_{r,q})$ which to lowest-order provides cross-Kerr like coupling $\phi_{a,b}^2\phi_{r,q}^2$. However, these internal modes are extremely high frequency (high E_C from low junction capacitance and high E_J from large individual JJs used in the chain), e.g. > 35 GHz, so they can be safely taken to be frozen in the ground state where their cross-Kerr interaction with the resonator and qubit modes a, b can be ignored. Furthermore, we can choose to fabricate the junctions in the array to have reasonable capacitances (e.g. about 5 fF such as in Eq. (S12)) such that these internal modes have $E_J/E_C > 50$ so they can really be viewed as extremely high frequency transmons. This means the internal modes have steady frequencies and do not impart charge noise to the resonator and qubit modes (that they are nonlinearly coupled with). Therefore, we can safely ignore these internal modes, i.e. assume $\cos{(\phi_{a,b})}\cos(\phi_{r,q}) \approx \cos{(\phi_{a,b})}$, which leads us to the simplified form of the potential energy used in main text.

Derivation of photon-enhanced squeezing

One non-ideal quarton coupling effect is the addition of negative self-Kerrs and cross-Kerr to the qubit and resonator due to photon number dependent (correlated) squeezing terms of the form $(b^{\dagger 2} + b^2)a^\dagger a$ and $(a^{\dagger 2} + a^2)b^\dagger b$. These terms originate from the same Hamiltonian coupling term $\phi_a^2\phi_b^2$ that gives the ideal cross-Kerr coupling, so they are unavoidable. We can model the effects of these terms by looking at a toy model of two harmonic oscillators coupled by only one of these terms:

$$H_{toy} = \omega_a a^\dagger a + \omega_b b^\dagger b + \zeta(b^{\dagger 2} + b^2)a^\dagger a \tag{S14}$$

where we can assume $\frac{\zeta}{\omega_b} \ll 1$ (generally true for quarton coupling). We can perform a Schrieffer-Wolff transformation on this Hamiltonian with the unitary

$$S = \exp\left(\frac{1}{2}a^\dagger a(z^* b^2 - z b^{\dagger 2})\right) \tag{S15}$$

and choose $z = re^{i\theta}$ to be real with $\theta = 0$ for simplicity. By setting the coefficients of the off-diagonal b^2 terms in $\tilde{H} = SH_{toy}S^\dagger$ to 0, we obtain the condition

$$\tanh(2ra^\dagger a) = \frac{2\zeta}{\omega_b}a^\dagger a \tag{S16}$$

and with $\zeta/\omega_b \ll 1$, this is satisfied with $r = \zeta/\omega_b$. Then by expanding our transformed Hamiltonian $\tilde{H}$ to second order in ζ, we have

$$\begin{aligned}\tilde{H} = &\left(\omega_a - \frac{2\zeta^2}{\omega_b}\cosh(2ra^\dagger a)\right)a^\dagger a + \omega_b b^\dagger b\cosh(2ra^\dagger a) + \omega_b\sinh^2(ra^\dagger a)\\ &-\frac{4\zeta^2}{\omega_b}a^\dagger ab^\dagger b\cosh(2ra^\dagger a) - \frac{2\zeta^2}{\omega_b}a^{\dagger 2}a^2\cosh(2ra^\dagger a) - \frac{4\zeta^2}{\omega_b}a^{\dagger 2}a^2 b^\dagger b\cosh(2ra^\dagger a)\end{aligned} \tag{S17}$$

and expanding to second order in r and taking only the 4 wave mixing terms or lower, we have the transformed Hamiltonian

$$\tilde{H} \approx \left(\omega_a - \frac{\zeta^2}{\omega_b}\right)a^\dagger a + \omega_b b^\dagger b - \frac{2\zeta^2}{\omega_b}a^\dagger ab^\dagger b - \frac{\zeta^2}{\omega_b}a^\dagger a^\dagger aa \tag{S18}$$

which adds negative cross-Kerr $\frac{2\zeta^2}{\omega_b}$ and negative self-Kerr $\frac{2\zeta^2}{\omega_b}$ to mode a. This result holds for coupling via the other correlated squeezing term $(a^{\dagger 2} + a^2)b^\dagger b$ also, which analogously adds negative cross-Kerr $\frac{2\zeta^2}{\omega_a}$ and negative self-Kerr $\frac{2\zeta^2}{\omega_a}$ to mode b. Note that the factor of 2 in the self-Kerr originates from the self-Kerr K being $\frac{K}{2}a^\dagger a^\dagger aa$.

In typical quarton coupling circuits, the correlated squeezing term has magnitude ζ directly proportional to the ideal cross-Kerr χ:

$$\zeta = \chi/2 \tag{S19}$$

since they both originate from the $\phi_a^2\phi_b^2$ coupling Hamiltonian.

Summary of Kerr effects in quartonic readout circuit

Kerr nonlinearity relative to linear inductance is generally weakened by chaining together more JJs in series (*27*). If we generalize the Hamiltonian in the main text to allow for any number of chained JJs in each mode, we can get a Hamiltonian:

$$\begin{aligned}\hat{H} = 4E_{Ca}\hat{n}_a^2 + 4E_{Cb}\hat{n}_b^2 - n_{Ja}E_{Ja}\cos\left(\frac{\hat{\phi}_a}{n_{Ja}}\right) - n_{Jb}E_{Jb}\cos\left(\frac{\hat{\phi}_b}{n_{Jb}}\right) \\ +\alpha E_J\cos(\hat{\phi}_a - \hat{\phi}_b + \tilde{\phi}) - n_S E_J \cos\left(\frac{\hat{\phi}_a - \hat{\phi}_b}{n_S}\right)\end{aligned} \tag{S20}$$

Tables S1-2 summarize the various sources of self- and cross-Kerr effects in this generalized quartonic readout setup, along with their expected analytic scalings. Two main causes of self- and cross-Kerr (other than inherent JJ self-Kerr) are the bare mode quarton Kerr effects derived in (*27*) and the correlated squeezing Kerr effects as derived above. A main goal in parameter optimization is to have the self-Kerr (and higher level nonlinearity) in the resonator be net zero while maintaining high (hundreds of megahertz) self-Kerr in the qubit and cross-Kerr between the qubit and the resonator. Choices of the number of series junctions $\{n_S, n_{Ja}, n_{Jb}\}$ generally have an influence on nonlinearity and therefore decisions should be made prudently to help achieve the goals in optimization. Generally speaking, larger n_S simply increases E_Q relative to αE_J (*27*), whereas larger n_{Ja}, n_{Jb} drastically decreases the intrinsic negative self-Kerr in mode a, b. This is most relevant for the resonator mode a, which we must linearize (achieve net zero self-Kerr). To that end, n_{Ja} is an exceptionally valuable tuning knob, and we have opted for $n_{Ja} = 2$ in the main text for the particular combination of resonator and qubit frequency we were working with. Other parameter ranges could certainly benefit from different n_{Ja}.

Details on Fock Basis Treatment

A good basis is essential for effectively simulating coupled quantum systems. As described in Methods, we split our Hamiltonian into three terms to isolate the coupling terms:

$$H = H_a + H_b + H_{coup} \tag{S21}$$

and in order to accurately and efficiently represent the circuit eigenstates and their associated eigenenergies, we want to find a Fock basis that well represents the eigenstates of H_j for each mode $j = a, b$. We aim to maximize

$$\sum_{k=0}^{N_j} |\langle k_j | e_{k,j} \rangle|^2 \tag{S22}$$

where $|e_{k,j}\rangle$ is the kth eigenstate of H_j. This translates to the general problem with finding a Fock basis for a potential $U(\phi)$ that isn't necessarily quadratic. One approach is to sweep possible values of $\phi_{zpf} = 1/(2n_{zpf})$ and search for the maximum overlap as seen in Fig. S2. There, we also demonstrate the performance of two analytical heuristics, first by minimizing the magnitude of the $a^\dagger a^\dagger$ term coefficient, and by minimizing the coefficient of $a^\dagger a$ terms. We may choose to implement analytical heuristics for computational efficiency.

As seen in Fig. S2, minimizing the normal ordered $a^\dagger a$ coefficients tend to give high average overlaps between our basis and H_j eigenstates. Since the Fock basis is a complete basis, the final eigenenergies of H should be independent of the exact choices of the ϕ_{zpf} values, so the exact optimality of our ϕ_{zpf} values is unimportant.

Our Fock basis cannot be perfect due to the nonlinear nature of our circuit and the terms that do not preserve photon numbers (e.g. a^2). Additionally, one may notice that each bare Hamiltonian H_j includes ϕ_{zpf_k} values from other modes $k \neq j$. Thus the optimization of each basis isn't completely independent. However, this ultimately doesn't affect our relevant full system eigenenergies, and with sufficiently high overlap probabilities, we can label the eigenstates without ambiguity.

In decomposing H into $H_a + H_b + H_{coup}$ to construct our bases, we normal order all the creation and annihilation operators to analytically separate terms into their respective partitions. Normal ordering is important for representing the excitations relative to the ground state, and is also important for numerical simulations, since terms such as $aa^\dagger$ in a finite Hilbert space will incorrectly map the highest Fock state to 0. This framework also helps with finding approximate analytical quantities, where we expect to see the terms $\frac{K_b}{2} b^\dagger b^\dagger bb \in H_b$ or $2\chi a^\dagger a b^\dagger b \in H_{coup}$. This is another reason why we aim to optimize our Fock basis, so that the creation and annihilation operators can more closely represent transitions between adjacent eigenstates.

Master equation simulation

Given full parameters to the time independent Hamiltonian $\hat{H}_0$, we use QuTiP (*33*) to solve for the eigenstates and eigenenergies in the Fock basis, using a sufficiently large Hilbert space dimension for each subsystem (labelled N_a, N_b) such that the eigenstates of interest satisfy the commutator relations $[\hat{a}, \hat{a}^\dagger] = [\hat{b}, \hat{b}^\dagger] = 1$ numerically. In practice, this requires a large total Hilbert space size $N_a \times N_b \approx 32 \times 32$, so in order to efficiently perform the time domain master equation simulations, we change to a truncated set of eigenstates $\{|e_i\rangle\}$ as our basis ($\hat{H}_{ij} = \langle e_i | \hat{H} | e_j \rangle$). The truncated eigenstates are labelled by their max overlap with the bare

states $|ij\rangle$, and we choose the truncated eigenstates $|e_i\rangle$ labelled with $i < i^*, j < j^*$ for imposed thresholds i^*, j^*. The thresholds are iteratively increased until the dynamics (c.f. Fig. 3BC in main text) converge, with typical values of $(i^*, j^*) \approx (10, 8)$.

Following standard treatment (*7*), we model the resonator drive through the coupling capacitor as the time dependent operator $\hat{H}_d(t) = \varepsilon(t)\hat{n}_0 = \varepsilon(t) \times i(\hat{a}^\dagger - \hat{a})$. Then, we use the Linblad-form master equation for the system's density matrix ρ:

$$\dot{\rho} = -i\left[\hat{H}_0 + \hat{H}_d(t), \rho\right] + \sum_k \kappa_k \mathcal{D}[\hat{d}_k]\rho \tag{S23}$$

where the k-indexed dissipators $\mathcal{D}(\hat{d}_k)\rho = d_k \rho d_k^\dagger - \frac{1}{2}(d_k^\dagger d_k \rho + \rho d_k^\dagger d_k)$ and their respective rates κ_k are found by (following (*24*)):

$$\sqrt{\kappa_k}\hat{d}_k = \sum_{i,j>i} \sqrt{\kappa_{\text{eff},ij}} \left| \langle e_j| (\hat{a}^\dagger - \hat{a}) |e\rangle_i \right| |e_i\rangle \langle e_j| \tag{S24}$$

Note that we are explicitly choosing a zero temperature bath which can only cause transitions from high to low ($j > i$) energy eigenstates $|e_j\rangle \to |e_i\rangle$. Furthermore, we define independent baths indexed by k, each coupled to the k'th set of eigenstate transitions $\{\omega_k\}$ that have overlapping line width (*24*). Eigenstate $j \to i$ transitions with frequencies $\omega_{ji} = \omega_j - \omega_i$ are considered to have overlapping line widths if they satisfy $|\omega_{ji} - \omega_{j'i'}| \leq c * \kappa$ (for some order unity constant c). In summary:

$$|e_i\rangle \langle e_j| \in \hat{d}_k \text{ iff } |\omega_{ji} - \omega_{j'i'}| \leq c\,\kappa \;\text{ for } \omega_{j'i'} \in \{\omega_k\} \tag{S25}$$

This is physically important as eigenstate transitions within about κ in frequency are correlated in their coupling to the same bath mode (*24*), and independent of bath modes coupling transitions $\gg \kappa$ away. In practice, the labelling in Eq. (S25) above is done via a density-based clustering algorithm (e.g. DBSCAN) on an array of all the allowed eigenstate transitions $\{\omega_{ji}\}$, to identify each of the k baths. The results are shown in Fig. S3, where a threshold rate of 10 kHz was set to discard dissipations too slow to affect our $O(10)$ ns time simulations. Fig. S3's column 2 panels repeat plots in column 1 but with opacity of lines set in proportion to the transition's $\kappa_{\text{eff},ij}$. This shows that the rates are dominated by the eigenstate transitions that most resembles resonator single photon loss, which are the transitions we monitor in readout measurement. We index this bath by k^* and will use it as the monitored operator in the subsequent stochastic master equation simulation.

Note that in Eq. (S24), we use a realistic effective decay rate $\kappa_{\text{eff},ij}$ that is weighted by the coupling frequency dependence ($\kappa \propto \omega_{ji}^2$) (*7*) and the Purcell filtering response ($\kappa \propto [1 + (2(\omega_{ji} - \omega_r)/\kappa_f)^2]^{-1}$) (*48*). The effect of the Purcell filter's suppression of unwanted bath coupling far from the drive frequency is clearly visible in column 2 of Fig. S3. For the readout parameters in the main text, the use of the Purcell filter improves QND fidelity from 99.5% to 99.96%. We also chose a nominal value of $c = 1$ in Eq. (S25), but the results of the Linblad-form master equation simulation seem robust to a range of c values we tested (around 0.5 -2).

Having constructed our Lindblad dissipators, we can use the Lindblad-form master equation of Eq. (S23) to find the average dynamics of the resonator-qubit during readout, producing plots like Fig. 3 in the main text. This is sufficient for determining properties such as QND fidelity. However, to rigorously obtain readout fidelity, we instead simulate measurement trajectories using the stochastic master equation (*33*):

$$d\rho(t) = d_1\rho dt + d_2\rho dW \tag{S26}$$

$$d_1\rho = -i\left[\hat{H}_0 + \hat{H}_d(t), \rho\right] + \sum_k \kappa_k \mathcal{D}[\hat{d}_k]\rho \tag{S27}$$

$$d_2\rho = S_{k^*}\rho(t) + \rho(t)S_{k^*}^\dagger - \mathrm{tr}\left(S_{k^*}\rho(t) + \rho(t)S_{k^*}^\dagger\right)\rho(t) \tag{S28}$$

As mentioned previously, the k^* indexed monitored operator S_{k^*} represents the resonator transitions for the different qubit states (blue lines in Fig. S3). By construction, the stochastic master equation will produce the same average dynamics as the deterministic master equation, while providing realistic measurement trajectories. Since the starting state of our readout simulation is always a pure state (eigenstate of qubit 0,1), we use QuTiP's stochastic Schrodinger equation solver $ssesolve$ (*33*). Iteratively choosing QuTiP stochastic solver parameters for convergence, we find that about $ntraj$ ≈12,800 trajectories, $nsubsteps$ ≈200 substeps for a given time step of $1/(5\omega_d)$ worked well. The resulting numerical heterodyne measurement trajectories are demodulated by multiplying a phase $\exp(i\omega_d t)$ and integrated to generate Fig. 4 of main text, from which SNR and readout fidelity can be obtained.

Approximations in the master equation

Master equations are derived with (*45*) many approximations such as the Markovian or Born approximations. Our quartonic readout designs use very large $\kappa/2\pi = 300$ MHz, so it is worth checking if these approximations are still valid.

In the literature, Purcell filters routinely have quality factors of about 30 and many hundreds of MHz of decay rate κ_f (e.g. (*12*) $\kappa_f/2\pi = 310$ MHz and (*31*) $\kappa_f/2\pi = 224$ MHz), and the standard Lindblad master equation was successful in reproducing experimental results (*48*). Therefore, the standard approximations used to derive the master equation such as the Markovian and Born approximations should still be valid in for our design with similar $\kappa/2\pi = 300$ MHz and $Q = \omega_a/\kappa \approx 50$.

However, our Purcell filter has much larger $\kappa_f = 4\kappa = 2\pi \times 1.2$ GHz decay rate, corresponding to a low quality factor of $Q \approx 13$. Although this is the same order of magnitude as experimentally demonstrations (*12, 31*), it is an open question whether the coupling of this Purcell filter to the bath is strong enough to violate the master equation's underlying assumptions. Therefore, we have opted to not include the Purcell filter as part of the quantum system in the master equation simulations, but have instead treated it more classically as a filtering function on the resonator's decay to the bath ($\kappa \propto [1 + (2(\omega_{ji} - \omega_r)/\kappa_f)^2]^{-1}$). Note also that we chose a small ratio of $\kappa_f/\kappa = 4$ but this has been experimentally demonstrated (*41*).

Decoherence analysis

Purcell Decay and Shot Noise Dephasing

Ideally, any readout scheme should not worsen the qubit's decoherence (T_1, T_2). Here, we examine how common readout-induced decoherence channels such as Purcell decay (*25*) and thermal shot noise dephasing (*49,50*) can be suppressed in the proposed quarton-coupler readout scheme.

Without the Purcell filter, the readout resonator is coupled via its (normalized) charge operator $\hat{n}_0 := i(\hat{a}^\dagger - \hat{a})$ to the readout environment (or bath) with frequency dependent rate $\kappa(\omega)$, and the qubit is coupled via the tilted quarton to the readout resonator, the eigenstates transitions of the system $|e_j\rangle \to |e_i\rangle$ are effectively coupled to the bath with rate $\Gamma_{ji} = \kappa(\omega_{ji})|\langle e_j|\hat{n}_0|e_i\rangle|^2$ (*24*). So for qubit eigenstates $\{|e_j\rangle, |e_i\rangle\} = \{|1\rangle, |0\rangle\}$, the relaxation rate is enhanced by the Purcell decay rate $\Gamma_P \equiv \Gamma_{10}$. It is worth emphasizing that quarton-coupler's zero Purcell decay with large cross-Kerr can be achieved *without* Purcell filters, in contrast to state-of-the-art dispersive readout where Purcell decay cannot be avoided without Purcell filters due to the underlying linear coupling required for cross-Kerr.

In dispersive readout, large Γ_P is typically suppressed by adding Purcell filters (*15, 31, 51–53*) that minimize qubit $\kappa(\omega_q) \to 0$ while keeping resonator $\kappa(\omega_r)$ unchanged. While a Purcell filter is not required for quarton-coupler readout to eliminate Purcell decay at $\kappa(\omega_q)$, it is nevertheless beneficial for the related but different purpose of suppressing unwanted decay at other eigenstate transitions (see supplementary text for details). Purcell filtering is highly compatible with quarton-coupler readout since transition frequencies to filter are usually many gigahertz away from the resonator frequency ω_r we wish to preserve. State-of-the-art bandpass Purcell filters with quality factors $Q_f \approx 10$ can be used, which is sufficiently low (*41*) compared to the quality factor of the readout resonator $Q_r = \omega_r/\kappa(\omega_r) \approx 50$ for the Purcell filter to operate ideally (*48*).

Qubit dephasing from shot noise of thermal photons in the resonator is another important source of decoherence that increases with κ (for fixed χ/κ) (*7, 44*). For an average thermal photon number $\bar{n}_t$, the shot noise dephasing rate is given by (*49*)

$$\gamma_m = \frac{\bar{n}_t(\bar{n}_t + 1)(2\chi)^2}{\kappa(\omega_r)}. \tag{S29}$$

We can substantially reduce γ_m in quartonic readout by leveraging both the frequency-tunability and the higher resonant frequency of quarton coupled readout resonators. The frequency-tunability of quarton coupled readout resonators stems from the use of a lumped-element transmon-like mode for readout, with inductance provided by JJs which can be replaced with SQUIDs that can be in-situ flux-tuned (*54*). This allows for potential schemes that flux-tune the readout resonator into a very low κ frequency band of the readout transmission line (e.g. protected by a filter) to reduce γ_m before readout, and flux-tune the resonator back to the desired κ frequency during readout. We note that the key operating principle of lowering κ by flux-tuning a resonator with JJs has been experimentally demonstrated in the literature (*12*). Such active flux-tuning

schemes may also be replaced by a more hardware-efficient, passive scheme of shot noise protection in the form of a well-thermalized (*50, 55*) high frequency readout resonator (*12*). Unlike dispersive readout, quartonic readout performance does not explicitly depend on qubit-resonator frequency detuning, so the quarton coupled readout resonator can be made very high frequency (e.g.$\approx$ 18.5 GHz in Table 1) relative to current transmon qubits and dispersive readout resonators (typically 3-8 GHz (*8*)). Since γ_m scales directly with the average thermal photon population following Bose-Einstein statistics: $\bar{n}_t(T) = [\exp(\hbar\omega_r/(k_B T)) - 1]^{-1}$, a marginally higher resonator frequency $\omega_r = 2\pi \times 12.5$ GHz with state-of-the-art thermalization (effective temperature $T = 45$ mK (*55*)) can have 10^2 times lower $\bar{n}_t$ and thus 10^2 times lower γ_m compared to a state-of-the-art dispersive readout resonator $\omega_r = 2\pi \times 8$ GHz, thereby nullifying the impact of 10^2 times larger κ. In fact, with even larger $\omega_r/2\pi \gg 12.5$ GHz easily achievable for quartonic readout, the γ_m increase from larger κ may be more than offset by many orders of magnitude lower $\bar{n}_t$, making the quartonic readout's γ_m net lower than state-of-the-art dispersive readout's. However, we note that the highest frequency readout resonator with reported thermal photon population in the literature is 9.8 GHz with $\bar{n}_t = 4 \times 10^{-4}$ (*12*), whereas our proposed readout resonator frequency will be about 18.5 GHz. Thermalizing microwave components to the mixing chamber stage of a dilution refrigerator remains an active area of research (*50, 55*).

Relaxation from Quasiparticles

Non-equilibrium quasiparticles are a well-known source of loss for superconducting qubits (*7, 8*). Quasiparticle decay of a superconducting qubit generally follows (*56*)

$$\Gamma_{qp} = \left| \langle 0| \sin\frac{\hat{\phi}}{2} |1\rangle \right|^2 \frac{8E_J}{\pi\hbar} x_{\mathrm{qp}} \sqrt{\frac{2\Delta}{\hbar\omega_q}}, \tag{S30}$$

where ω_q is the qubit frequency, and E_J and $\hat{\phi}$ are the Josephson energy and the phase operator for the junction that the quasiparticles tunnel through. Applying Eq. (S30) above to each junction in our circuit, and using state-of-the-art values of quasiparticle density $x_{qp} = 5 \times 10^{-9}$ (*57*) and superconducting gap of aluminum $2\Delta/h = 82$ GHz (*7*), we estimate $T_{1,qp} = 1/\Gamma_{qp}$ to be approximately 0.46 ms for the transmon qubit parameters we used for readout simulation. These values are about an order of magnitude worse than the inherent qubit $T_{1,qp}$ without quarton coupling as a direct consequence of the quarton adding additional high Josephson energy $\sim E_Q$ Josephson junctions to the qubit. We chose parameters of high E_Q (~ 5 times higher than intrisinc transmon E_J) because we opted for very high cross-Kerr $\chi \propto E_Q$ (*27*) to demonstrate ultrafast readout. Quasiparticle loss mitigation techniques may be applied to suppress $T_{1,qp}$ without sacrificing readout speed. Examples of mitigation techniques include quasiparticle traps (*58*) and shielding (*59*).

Decoherence from Flux Noise

Flux noise is a well-known decoherence channel with "quasi-universal" noise power spectrum (*8*): $S_\Phi(\omega) = A_\Phi^2 \left(\frac{2\pi\times 1\text{ Hz}}{\omega}\right)^{\gamma_\Phi}$ with $\gamma_\Phi \approx 0.8 - 1.0$ and $A_\Phi^2 \approx (1\,\mu\Phi_0)^2/\text{Hz}$. Qubit relaxation from flux noise follows (*8*):

$$\Gamma_{1,\Phi} = \left|\langle 0| \frac{\partial H}{\partial \Phi} |1\rangle\right|^2 S_\Phi(\omega_q), \tag{S31}$$

where to capture the time dependence explicit in the $\frac{\partial H}{\partial \Phi}$ term, it is important to follow the approach of (*60*) and write an "irrotational" Hamiltonian for our system, eliminating the term proportional to the time derivative of the flux noise. In our analysis, we assume that the flux noise through the two loops is independent, so we can analyze noise in the two loops separately. This means we only ever have one time dependent flux, simplifying the analysis.

We define branch fluxes and capacitances as in Fig. S4. For time-dependent flux $\tilde{\phi} = 2\pi\frac{\Phi}{\Phi_0}$ in the ground loop, this yields

$$\begin{aligned}\hat{H}_{irr} &= T - 2E_{Ja}\cos\left(\frac{1}{2}(\phi_a - \frac{C_a^{-1}}{\sum_i C_i^{-1}}\tilde{\phi})\right) - E_{Jb}\cos\left(\phi_b + \frac{C_b^{-1}}{\sum_i C_i^{-1}}\tilde{\phi}\right) \\ &+E_{J\alpha}\cos\left(\phi_b - \phi_a - \frac{C_Q^{-1}}{\sum_i C_i^{-1}}\tilde{\phi}\right) - 2E_{Js}\cos\left(\frac{1}{2}(\phi_b - \phi_a - \frac{C_Q^{-1}}{\sum_i C_i^{-1}}\tilde{\phi}\right),\end{aligned} \tag{S32}$$

where T is the usual capacitive energy term and $C_Q = C_s + C_\alpha$. Similarly, if the time-dependent noise $\tilde{\phi}$ instead threads the quarton loop, we obtain

$$\begin{aligned}\hat{H}_{irr} &= T - 2E_{Ja}\cos\left(\frac{1}{2}(\phi_a + A\tilde{\phi})\right) - E_{Jb}\cos\left(\phi_b + B\tilde{\phi}\right) \\ &+E_{J\alpha}\cos\left(\phi_b - \phi_a + (B-A)\tilde{\phi}\right) - 2E_{Js}\cos\left(\frac{1}{2}(\phi_b - \phi_a + (B-A+1)\tilde{\phi}\right),\end{aligned} \tag{S33}$$

with

$$A = \frac{C_b C_s}{2C_a C_b + (C_a + C_b)(C_\alpha + C_s)}$$
$$B = \frac{C_a C_s}{2C_a C_b + (C_a + C_b)(C_\alpha + C_s)}$$

We can then compute the T_1 decoherence contribution of each loop as

$$\Gamma_\Phi = \left|\langle 0| \frac{\partial H}{\partial \Phi} |1\rangle\right|^2 S_\Phi(\omega_q) \tag{S34}$$

with $S_\Phi(\omega) = A_\Phi^2 \left(\frac{2\pi\times 1\text{ Hz}}{\omega}\right)^{\gamma_\Phi}$. We use $A_\Phi^2 = (1\,\mu\Phi_0)^2/\text{Hz}$ and $\gamma_\Phi = 1$ for the quarton loop (*8*). Since we anticipate the ground loop being larger, we increase the flux noise amplitude

to $A_\Phi^2 = (5\,\mu\Phi_0)^2/\mathrm{Hz}$, corresponding to a 25x increase in loop perimeter (*61*). Applying Eq. (S31) to each loop and adding the resulting $\Gamma_{1,\Phi}$ together, we get a $T_{1,\Phi}$ of 2.1 ms for the parameters in Table 1.

When estimating the pure dephasing caused by flux noise, we choose to simulate an echo measurement rather than a Ramsey measurement. A Ramsey measurement would be very sensitive to the length of numerical time series used (as a longer time series includes more low frequency noise). We simulate the echo measurement by first generating a long ($\approx$ 3 hour) time series with power spectral density given by $S_\Phi(\omega)$, and then dividing it into many short time series. This avoids artificially filtering out low frequency noise that could affect the echo measurement. For each flux noise time series, we compute the qubit frequency at each time and integrate it (with an added sign flip in the middle) to compute the dephasing of a typical echo sequence. We then average the resulting echo sequence over all the time series. Again assuming noise in each loop is independent and fitting an exponential decay to the dephasing from each loop, we obtain a combined $T_{2,\Phi}$ of 11.4 ms (see supplementary text).

Relaxation from Dielectric Loss

Superconducting qubits suffer relaxation from dielectric loss at rate (*62*):

$$\Gamma_{\text{diel}} = \frac{\hbar\omega_q^2}{8E_C Q_{\text{diel}}}|\langle 0|\hat{\phi}|1\rangle|^2\left[\coth\left(\frac{\hbar\omega_q}{2k_B T}\right)+1\right]. \tag{S35}$$

Using experimental $Q_{\text{diel}} = 11.7\times 10^6$ value (*63*) with our quarton coupled qubit frequencies and matrix elements, we compute $T_{1,\text{diel}} = 1/\Gamma_{\text{diel}} \approx 285\ \mu$s for the transmon qubit in Table 1. Unlike all previous decoherence calculations, dielectric loss does not depend explicitly on the unusually large parameters such as E_Q or κ in quartonic readout, so as expected, $T_{1,\text{diel}}$ is essentially unaffected by the quarton.

Experimental feasibility details

We perform Monte Carlo analysis of the quartonic circuit, where we assume that each Josephson energy in the circuit (E_{Ja}, E_{Jb}, αE_J, E_J) follows a normal distribution with a relative standard deviation of 1.1%. This degree of variation has previously been demonstrated on a $10\,\mathrm{mm}\times 10\,\mathrm{mm}$ chip (*64*). The simulated yield is assessed based on the criteria of resonator dispersion below $50\,\mathrm{MHz}$ and QND fidelity exceeding 99%. Over 6000 simulation runs, we obtain a yield of 96.0%. See Fig. S5 for histograms of the resonator dispersion, QND fidelity, cross Kerr, and qubit anharmonicity. The QND fidelity is determined using the full master equation simulations.

Another experimental imperfection is deviation from ideal flux bias. We can model the effect of deviations $\Delta\phi$ of the flux bias $\Phi_{ext} = \frac{\Phi_0}{2\pi}(\pi+\Delta\phi)$ by varying the potential of one branch of the quarton $-\alpha E_J\cos(\hat{\phi}_a - \hat{\phi}_b + \pi + \Delta\phi)$. In the Fock basis, this is easier to simulate

as

$$U = -2E_{Ja}\cos(\hat{\phi}_a/2) - E_{Jb}\cos(\hat{\phi}_b) - 2E_J\cos\left(\frac{\hat{\phi}_a - \hat{\phi}_b}{2}\right) \\ +\alpha E_J\cos(\hat{\phi}_a - \hat{\phi}_b)\cos(\Delta\phi) - \alpha E_J\sin(\hat{\phi}_a - \hat{\phi}_b)\sin(\Delta\phi) \tag{S36}$$

and we can find the analytic QND fidelity and cross-Kerr (to predict readout speed) for small changes in $\Delta\phi$ as seen in Fig. S6. For deviations within 1% of a flux quantum, we still expect $> 99.9\%$ QND fidelity and have only small variations in χ.

Ionization / MIST analysis

Here, we examine our proposed quartonic readout system's susceptibility to the effect of ionization (*39*) (driven chaos (*21*)) or measurement-induced state transitions (MIST) (*20*) present in state-of-the-art dispersive transmon readout systems. Using the operating point in the main text, we perform branch analysis following the methodology of Ref. (*39*). This leads to Fig. S7 (c.f. Fig. 2a of Ref. (*39*)), which shows that both qubit states $|0\rangle, |1\rangle$ undergo ionization near the critical photon number of $N_r = 10$. This suggests that our proposed quartonic readout should use a readout coherent state with minimal Fock state $|n = 10\rangle$ population, which justifies our choice of readout coherent state $|\alpha = \sqrt{2}\rangle$ in the main text simulations.

We note that unlike most results in other sections which use the Fock basis, these ionization / MIST simulations are performed in the charge basis to include coupling to the unbound charge states (*39*). We also included gate charge n_g in the model but found no substantial change in the critical photon number with n_g of either qubit or resonator mode.

1 Supplementary Materials Figures

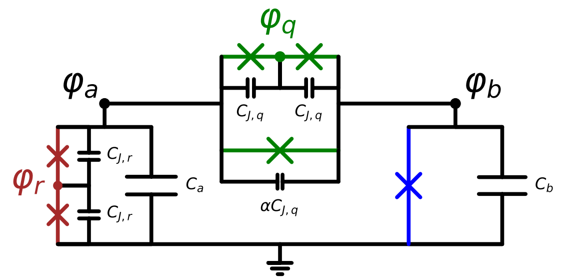

Fig. S1. Labeled nodes for full circuit Hamiltonian derivation. The internal quarton mode is included, and we assume half flux quantum bias in the quarton loop (and none elsewhere).

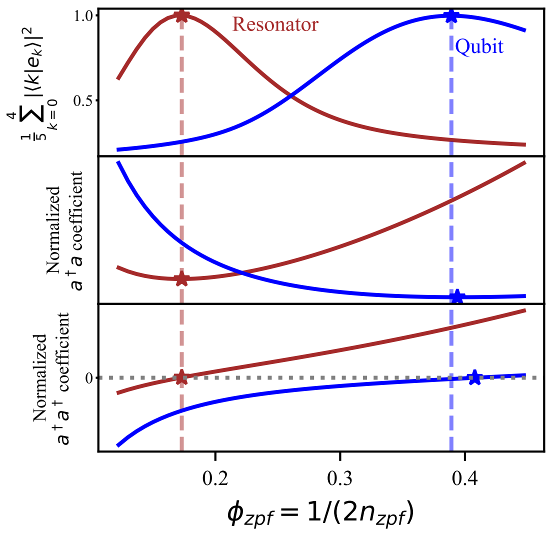

Fig. S2. Summary of ϕ_{zpf} heuristics. These are used to choose $\phi_{zpf} = 1/(2n_{zpf})$ values for representing eigenenergies in a nonlinear potential. The first metric finds the average overlap $\sum_{k=0}^{4} |\langle k_j|e_{k,j}\rangle|^2$ for each mode j. The heuristics of minimizing the coefficient of an unwanted $a^\dagger a^\dagger$ term and minimizing the first order energy $a^\dagger a$ term are displayed in comparison. The bare modes H_a, H_b are calculated with 8th order Taylor expansions of the original Hamiltonian and normal ordered with computational symbolic algebra.

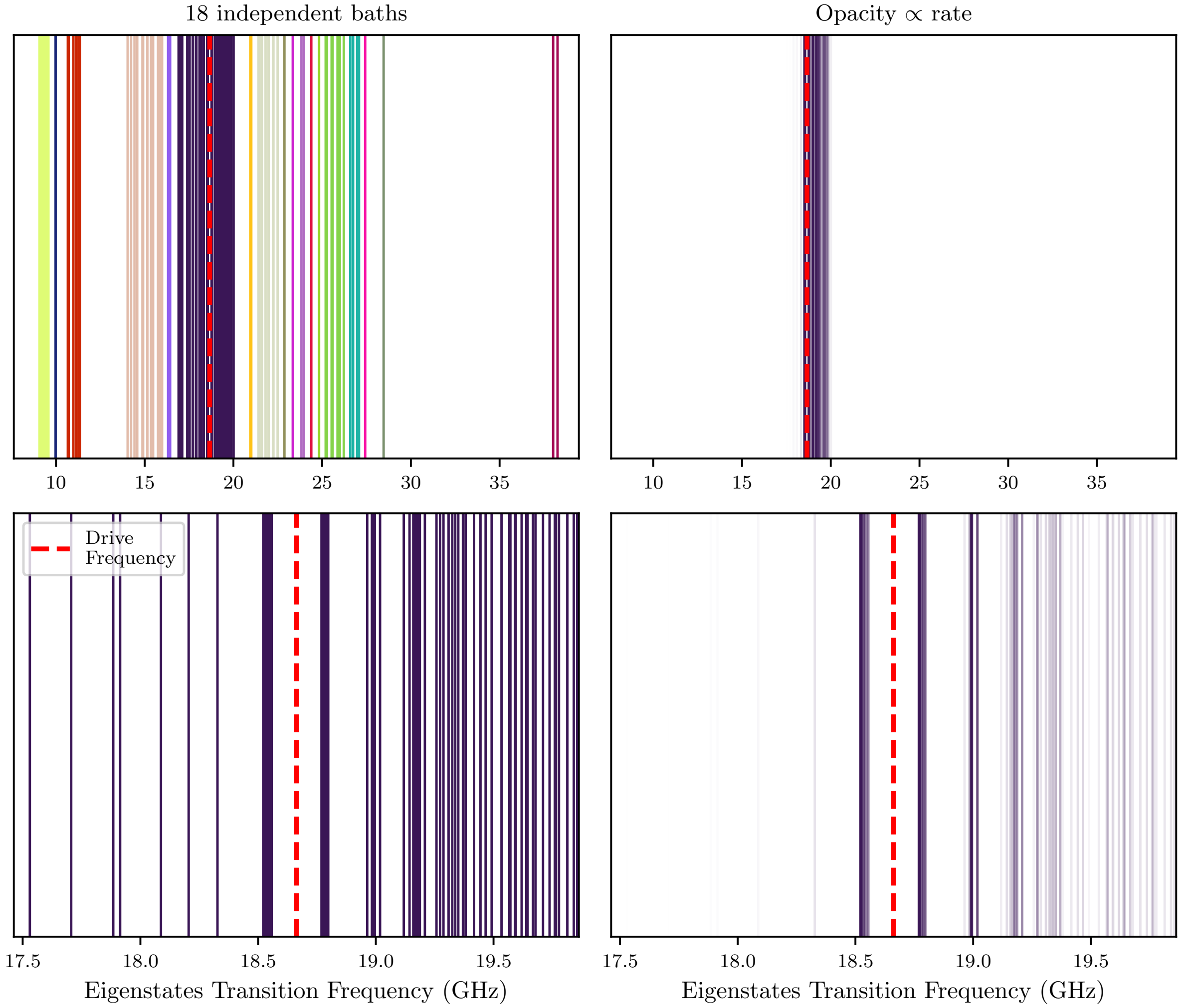

Fig. S3. Independent baths (different colors) found by density-based clustering algorithm (DBSCAN). Only the eigenstate transitions that most resembles resonator single photon loss have high rates (high opacity in column 2 plots). Row 2 plots are zoomed-in (around resonator and drive frequency) views of row 1 plots, showing the most important dissipation (blue) to be labelled k^* and used as monitored operator in stochastic master equation.

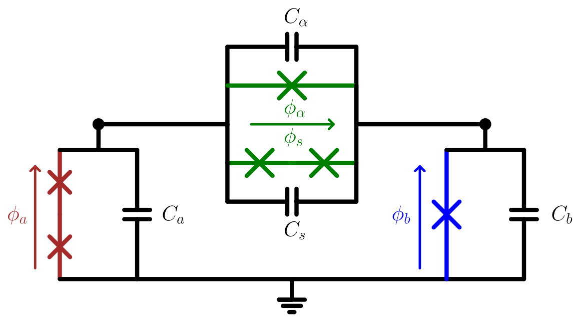

Fig. S4. Branch variables used in flux noise analysis

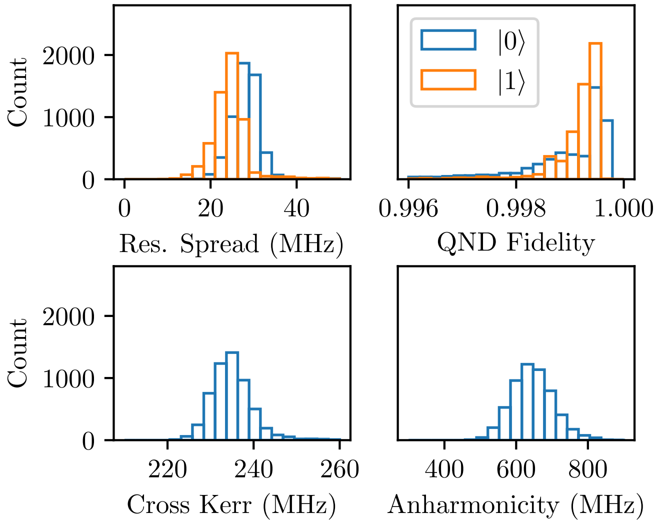

Fig. S5. Histograms of resonator dispersion, QND fidelity, cross Kerr, and anharmonicity over 6000 simulations, assuming that the Josephson energies of the quartonic circuit have relative standard deviation of 1.1%.

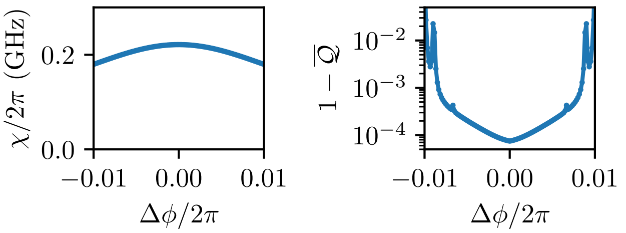

Fig. S6. Readout parameter sensitivity to deviation in flux bias $\Delta\phi$. Dependence of cross-Kerr χ and analytic QND infidelity ($1-\bar{\mathcal{Q}}$) on flux bias. Changing $\Delta\phi$ while holding all other parameters constant for the main text operating point illustrates the dependency of readout properties on flux bias deviation.

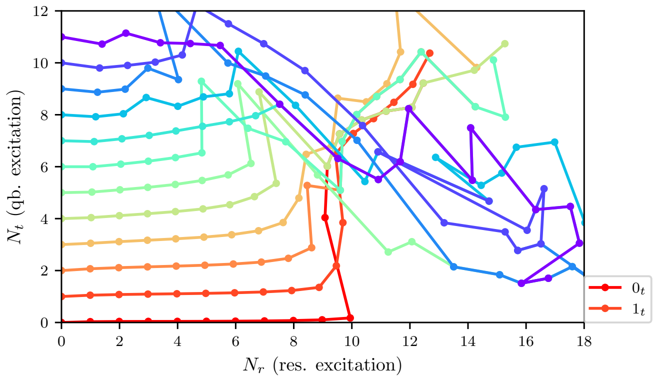

Fig. S7. Ionization / MIST analysis of quartonic readout. Following methodology of Ref. (*39*), branch analysis shows a critical readout photon number of $N_r = 10$.

2 Supplementary Materials Tables

Table S1. Summary of self-Kerr sources and analytic scalings.

	Mode a (resonator)	Mode b (qubit)
self-Kerr from internal JJ	$-\frac{1}{n_{Ja}^2} E_{C,a}$	$-\frac{1}{n_{Jb}^2} E_{C,b}$
self-Kerr from quarton coupler	$+\frac{E_Q}{E_{J,a}} E_{C,a}$	$+\frac{E_Q}{E_{J,b}} E_{C,b}$
self-Kerr from a 's squeezing $a^2 \left(b^\dagger b\right) +$ h.c.	0	$-\frac{\chi_{ab}^2}{2\omega_b} = -\frac{E_Q^2 E_{C,a}^{1/2}}{2E_{J,b} E_{J,a}^{3/2}} E_{C,b}$
self-Kerr from b 's squeezing $b^2 \left(a^\dagger a\right) +$ h.c.	$-\frac{\chi_{ab}^2}{2\omega_a} = -\frac{E_Q^2 E_{C,b}^{1/2}}{2E_{J,a} E_{J,b}^{3/2}} E_{C,a}$	0

Table S2. Summary of cross-Kerr sources and analytic scalings.

	Mode a - b (resonator-qubit)
cross-Kerr from quarton coupler	$+\chi_{ab} = 2E_Q \frac{\left(E_{C,a} E_{C,b}\right)^{1/2}}{\left(E_{J,a} E_{J,b}\right)^{1/2}}$
cross-Kerr from a's squeezing $a^2 \left(b^\dagger b\right) +$ h.c.	$-\frac{\chi_{ab}^2}{2\omega_b}$
cross-Kerr from b's squeezing $b^2 \left(a^\dagger a\right) +$ h.c.	$-\frac{\chi_{ab}^2}{2\omega_a}$

REFERENCES AND NOTES

1. S. Krinner, N. Lacroix, A. Remm, A. Di Paolo, E. Genois, C. Leroux, C. Hellings, S. Lazar, F. Swiadek, J. Herrmann, G. J. Norris, C. K. Andersen, M. Müller, A. Blais, C. Eichler, A. Wallraff, Realizing repeated quantum error correction in a distance-three surface code. *Nature* **605**, 669–674 (2022).

2. V. V. Sivak, A. Eickbusch, B. Royer, S. Singh, I. Tsioutsios, S. Ganjam, A. Miano, B. L. Brock, A. Z. Ding, L. Frunzio, S. M. Girvin, R. J. Schoelkopf, M. H. Devoret, Real-time quantum error correction beyond break-even. *Nature* **616**, 50–55 (2023).

3. C. H. Bennett, G. Brassard, C. Crépeau, R. Jozsa, A. Peres, W. K. Wootters, Teleporting an unknown quantum state via dual classical and Einstein-Podolsky-Rosen channels. *Phys. Rev. Lett.* **70**, 1895–1899 (1993).

4. L. Steffen, Y. Salathe, M. Oppliger, P. Kurpiers, M. Baur, C. Lang, C. Eichler, G. Puebla-Hellmann, A. Fedorov, A. Wallraff, Deterministic quantum teleportation with feed-forward in a solid state system. *Nature* **500**, 319–322 (2013).

5. J. Johnson, C. Macklin, D. Slichter, R. Vijay, E. Weingarten, J. Clarke, I. Siddiqi, Heralded state preparation in a superconducting qubit. *Phys. Rev. Lett.* **109**, 050506 (2012).

6. D. Ristè, J. G. van Leeuwen, H.-S. Ku, K. W. Lehnert, L. DiCarlo, Initialization by measurement of a superconducting quantum bit circuit. *Phys. Rev. Lett.* **109**, 050507 (2012).

7. A. Blais, A. L. Grimsmo, S. M. Girvin, A. Wallraff, Circuit quantum electrodynamics. *Rev. Mod. Phys.* **93**, 025005 (2021).

8. P. Krantz, M. Kjaergaard, F. Yan, T. P. Orlando, S. Gustavsson, W. D. Oliver, A quantum engineer’s guide to superconducting qubits. *Appl. Phys. Rev.* **6**, 021318 (2019).

9. F. Arute, K. Arya, R. Babbush, D. Bacon, J. C. Bardin, R. Barends, R. Biswas, S. Boixo, F. G. S. L. Brandao, D. A. Buell, B. Burkett, Y. Chen, Z. Chen, B. Chiaro, R. Collins, W. Courtney, A. Dunsworth, E. Farhi, B. Foxen, A. Fowler, C. Gidney, M. Giustina, R. Graff, K. Guerin, S.

Habegger, M. P. Harrigan, M. J. Hartmann, A. Ho, M. Hoffmann, T. Huang, T. S. Humble, S. V. Isakov, E. Jeffrey, Z. Jiang, D. Kafri, K. Kechedzhi, J. Kelly, P. V. Klimov, S. Knysh, A. Korotkov, F. Kostritsa, D. Landhuis, M. Lindmark, E. Lucero, D. Lyakh, S. Mandrà, J. R. McClean, M. McEwen, A. Megrant, X. Mi, K. Michielsen, M. Mohseni, J. Mutus, O. Naaman, M. Neeley, C. Neill, M. Y. Niu, E. Ostby, A. Petukhov, J. C. Platt, C. Quintana, E. G. Rieffel, P. Roushan, N. C. Rubin, D. Sank, K. J. Satzinger, V. Smelyanskiy, K. J. Sung, M. D. Trevithick, A. Vainsencher, B. Villalonga, T. White, Z. J. Yao, P. Yeh, A. Zalcman, H. Neven, J. M. Martinis, Quantum supremacy using a programmable superconducting processor. *Nature* **574**, 505–510 (2019).

10. A. Wallraff, D. I. Schuster, A. Blais, L. Frunzio, J. Majer, M. H. Devoret, S. M. Girvin, R. J. Schoelkopf, Approaching unit visibility for control of a superconducting qubit with dispersive readout. *Phys. Rev. Lett.* **95**, 060501 (2005).

11. T. Walter, P. Kurpiers, S. Gasparinetti, P. Magnard, A. Potočnik, Y. Salathé, M. Pechal, M. Mondal, M. Oppliger, C. Eichler, A. Wallraff, Rapid high-fidelity single-shot dispersive readout of superconducting qubits. *Phys. Rev. Appl.* **7**, 054020 (2017).

12. Y. Sunada, K. Yuki, Z. Wang, T. Miyamura, J. Ilves, K. Matsuura, P. A. Spring, S. Tamate, S. Kono, Y. Nakamura, Photon-noise-tolerant dispersive readout of a superconducting qubit using a nonlinear Purcell filter. *PRX Quantum* **5**, 010307 (2024).

13. M. Reed, L. DiCarlo, B. Johnson, L. Sun, D. Schuster, L. Frunzio, R. Schoelkopf, High-fidelity readout in circuit quantum electrodynamics using the Jaynes-Cummings nonlinearity. *Phys. Rev. Lett.* **105**, 173601 (2010).

14. N. Didier, J. Bourassa, A. Blais, Fast quantum nondemolition readout by parametric modulation of longitudinal qubit-oscillator interaction. *Phys. Rev. Lett.* **115**, 203601 (2015).

15. Y. Sunada, S. Kono, J. Ilves, S. Tamate, T. Sugiyama, Y. Tabuchi, Y. Nakamura, Fast readout and reset of a superconducting qubit coupled to a resonator with an intrinsic Purcell filter. *Phys. Rev. Appl.* **17**, 044016 (2022).

16. C. C. Bultink, B. Tarasinski, N. Haandbæk, S. Poletto, N. Haider, D. Michalak, A. Bruno, L. DiCarlo, General method for extracting the quantum efficiency of dispersive qubit readout in circuit QED. *Appl. Phys. Lett.* **112**, 092601 (2018).

17. N. Bergeal, F. Schackert, M. Metcalfe, R. Vijay, V. Manucharyan, L. Frunzio, D. Prober, R. Schoelkopf, S. Girvin, M. Devoret, Phase-preserving amplification near the quantum limit with a Josephson ring modulator. *Nature* **465**, 64–68 (2010).

18. C. Macklin, K. O'brien, D. Hover, M. Schwartz, V. Bolkhovsky, X. Zhang, W. Oliver, I. Siddiqi, A near–quantum-limited josephson traveling-wave parametric amplifier. *Science* **350**, 307–310 (2015).

19. K. Peng, M. Naghiloo, J. Wang, G. D. Cunningham, Y. Ye, K. P. O'Brien, Floquet-mode traveling-wave parametric amplifiers. *PRX Quantum* **3**, 020306 (2022).

20. D. Sank, Z. Chen, M. Khezri, J. Kelly, R. Barends, B. Campbell, Y. Chen, B. Chiaro, A. Dunsworth, A. Fowler, E. Jeffrey, E. Lucero, A. Megrant, J. Mutus, M. Neeley, C. Neill, P. O'Malley, C. Quintana, P. Roushan, A. Vainsencher, T. White, J. Wenner, A. N. Korotkov, J. M. Martinis, Measurement-induced state transitions in a superconducting qubit: Beyond the rotating wave approximation. *Phys. Rev. Lett.* **117**, 190503 (2016).

21. J. Cohen, A. Petrescu, R. Shillito, A. Blais, Reminiscence of classical chaos in driven transmons. *PRX Quantum* **4**, 020312 (2023).

22. M. Khezri, E. Mlinar, J. Dressel, A. N. Korotkov, Measuring a transmon qubit in circuit QED: Dressed squeezed states. *Phys. Rev. A* **94**, 012347 (2016).

23. J. Koch, M. Y. Terri, J. Gambetta, A. A. Houck, D. I. Schuster, J. Majer, A. Blais, M. H. Devoret, S. M. Girvin, R. J. Schoelkopf, Charge-insensitive qubit design derived from the Cooper pair box. *Phys. Rev. A* **76**, 042319 (2007).

24. F. Beaudoin, J. M. Gambetta, A. Blais, Dissipation and ultrastrong coupling in circuit QED. *Phys. Rev. A* **84**, 043832 (2011).

25. A. A. Houck, J. A. Schreier, B. R. Johnson, J. M. Chow, J. Koch, J. M. Gambetta, D. I. Schuster, L. Frunzio, M. H. Devoret, S. M. Girvin, R. J. Schoelkopf, Controlling the spontaneous emission of a superconducting transmon qubit. *Phys. Rev. Lett.* **101**, 080502 (2008).

26. R. Dassonneville, T. Ramos, V. Milchakov, L. Planat, E. Dumur, F. Foroughi, J. Puertas, S. Leger, K. Bharadwaj, J. Delaforce, C. Naud, W. Hasch-Guichard, J. J. García-Ripoll, N. Roch, O. Buisson, Fast high-fidelity quantum nondemolition qubit readout via a nonperturbative cross-Kerr coupling. *Phys. Rev. X* **10**, 011045 (2020).

27. Y. Ye, K. Peng, M. Naghiloo, G. Cunningham, K. P. O'Brien, Engineering purely nonlinear coupling between superconducting qubits using a quarton. *Phys. Rev. Lett.* **127**, 050502 (2021).

28. L. Neumeier, M. Leib, M. J. Hartmann, Single-photon transistor in circuit quantum electrodynamics. *Phys. Rev. Lett.* **111**, 063601 (2013).

29. M. Leib, P. Zoller, W. Lechner, A transmon quantum annealer: Decomposing many-body Ising constraints into pair interactions. *Quantum Sci. Technol.* **1**, 015008 (2016).

30. S. E. Nigg, H. Paik, B. Vlastakis, G. Kirchmair, S. Shankar, L. Frunzio, M. Devoret, R. Schoelkopf, S. Girvin, Black-box superconducting circuit quantization. *Phys. Rev. Lett.* **108**, 240502 (2012).

31. E. Jeffrey, D. Sank, J. Y. Mutus, T. C. White, J. Kelly, R. Barends, Y. Chen, Z. Chen, B. Chiaro, A. Dunsworth, A. Megrant, P. J. J. O'Malley, C. Neill, P. Roushan, A. Vainsencher, J. Wenner, A. N. Cleland, J. M. Martinis, Fast accurate state measurement with superconducting qubits. *Phys. Rev. Lett.* **112**, 190504 (2014).

32. R. Lescanne, M. Villiers, T. Peronnin, A. Sarlette, M. Delbecq, B. Huard, T. Kontos, M. Mirrahimi, Z. Leghtas, Exponential suppression of bit-flips in a qubit encoded in an oscillator. *Nat. Phys.* **16**, 509–513 (2020).

33. J. R. Johansson, P. D. Nation, F. Nori, QuTiP: An open-source Python framework for the dynamics of open quantum systems. *Comput. Phys. Commun.* **183**, 1760–1772 (2012).

34. K. Jacobs, D. A. Steck, A straightforward introduction to continuous quantum measurement. *Contemp. Phys.* **47**, 279–303 (2006).

35. D. T. McClure, H. Paik, L. S. Bishop, M. Steffen, J. M. Chow, J. M. Gambetta, Rapid driven reset of a qubit readout resonator. *Phys. Rev. Appl.* **5**, 011001 (2016).

36. B. Lienhard, "Machine learning assisted superconducting qubit readout," thesis, Massachusetts Institute of Technology, MA (2021).

37. L. Chen, H.-X. Li, Y. Lu, C. W. Warren, C. J. Križan, S. Kosen, M. Rommel, S. Ahmed, A. Osman, J. Biznárová, A. Fadavi Roudsari, B. Lienhard, M. Caputo, K. Grigoras, L. Grönberg, J. Govenius, A. F. Kockum, P. Delsing, J. Bylander, G. Tancredi, Transmon qubit readout fidelity at the threshold for quantum error correction without a quantum-limited amplifier. *npj Quantum Inf.* **9**, 26 (2023).

38. R. Hanai, A. McDonald, A. Clerk, Intrinsic mechanisms for drive-dependent Purcell decay in superconducting quantum circuits. *Phys. Rev. Res.* **3**, 043228 (2021).

39. M. F. Dumas, B. Groleau-Paré, A. McDonald, M. H. Muñoz-Arias, C. Lledó, B. D'Anjou, A. Blais, Unified picture of measurement-induced ionization in the transmon. arXiv:2402.06615 [quant-ph] (2024).

40. J. Hornibrook, J. Colless, A. Mahoney, X. Croot, S. Blanvillain, H. Lu, A. Gossard, D. Reilly, Frequency multiplexing for readout of spin qubits. *Appl. Phys. Lett.* **104**, 103108 (2014).

41. J. Heinsoo, C. K. Andersen, A. Remm, S. Krinner, T. Walter, Y. Salathé, S. Gasparinetti, J.-C. Besse, A. Potočnik, A. Wallraff, C. Eichler, Rapid high-fidelity multiplexed readout of superconducting qubits. *Phys. Rev. Appl.* **10**, 034040 (2018).

42. L. Stefanazzi, K. Treptow, N. Wilcer, C. Stoughton, C. Bradford, S. Uemura, S. Zorzetti, S. Montella, G. Cancelo, S. Sussman, A. Houck, S. Saxena, H. Arnaldi, A. Agrawal, H. Zhang, C. Ding, D. I. Schuster, The QICK (Quantum Instrumentation Control Kit): Readout and control for qubits and detectors. *Rev. Sci. Instrum.* **93**, 044709 (2022).

43. J. Wang, K. Peng, W. Van De Pontseele, K. Sliwa, P. Harrington, Y. Qiu, K. Serniak, J. Formaggio, W. Oliver, K. O'Brien; Project 8 neutrino mass experiment Team, K band Josephson traveling wave parametric amplifiers for neutrino mass measurement. *Bull. Am. Phys. Soc.* **2023**, Y71-010 (2023).

44. J. Gambetta, A. Blais, M. Boissonneault, A. A. Houck, D. I. Schuster, S. M. Girvin, Quantum trajectory approach to circuit QED: Quantum jumps and the Zeno effect. *Phys. Rev. A* **77**, 012112 (2008).

45. C. Gardiner, P. Zoller, *Quantum Noise: A Handbook of Markovian and Non-Markovian Quantum Stochastic Methods with Applications to Quantum Optics* (Springer Science & Business Media, 2004).

46. G. Viola, G. Catelani, Collective modes in the fluxonium qubit. *Phys. Rev. B* **92**, 224511 (2015).

47. D. Ding, H.-S. Ku, Y. Shi, H.-H. Zhao, Free-mode removal and mode decoupling for simulating general superconducting quantum circuits. *Phys. Rev. B* **103**, 174501 (2021).

48. E. A. Sete, J. M. Martinis, A. N. Korotkov, Quantum theory of a bandpass Purcell filter for qubit readout. *Phys. Rev. A* **92**, 012325 (2015).

49. P. Bertet, I. Chiorescu, G. Burkard, K. Semba, C. J. P. M. Harmans, D. P. DiVincenzo, J. E. Mooij, Dephasing of a superconducting qubit induced by photon noise. *Phys. Rev. Lett.* **95**, 257002 (2005).

50. F. Yan, D. Campbell, P. Krantz, M. Kjaergaard, D. Kim, J. L. Yoder, D. Hover, A. Sears, A. J. Kerman, T. P. Orlando, S. Gustavsson, W. D. Oliver , Distinguishing coherent and thermal photon noise in a circuit quantum electrodynamical system. *Phys. Rev. Lett.* **120**, 260504 (2018).

51. M. D. Reed, B. R. Johnson, A. A. Houck, L. DiCarlo, J. M. Chow, D. I. Schuster, L. Frunzio, R. J. Schoelkopf, Fast reset and suppressing spontaneous emission of a superconducting qubit. *Appl. Phys. Lett.* **96**, 203110 (2010).

52. N. T. Bronn, Y. Liu, J. B. Hertzberg, A. D. Córcoles, A. A. Houck, J. M. Gambetta, J. M. Chow, Broadband filters for abatement of spontaneous emission in circuit quantum electrodynamics. *Appl. Phys. Lett.* **107**, 172601 (2015).

53. N. T. Bronn, E. Magesan, N. A. Masluk, J. M. Chow, J. M. Gambetta, M. Steffen, Reducing spontaneous emission in circuit quantum electrodynamics by a combined readout/filter technique. *IEEE Trans. Appl. Supercond.* **25**, 1–10 (2015).

54. M. A. Castellanos-Beltran, K. Irwin, G. Hilton, L. Vale, K. Lehnert, Amplification and squeezing of quantum noise with a tunable Josephson metamaterial. *Nat. Phys.* **4**, 929–931 (2008).

55. Z. Wang, S. Shankar, Z. Minev, P. Campagne-Ibarcq, A. Narla, M. H. Devoret, Cavity attenuators for superconducting qubits. *Phys. Rev. Appl.* **11**, 014031 (2019).

56. G. Catelani, R. J. Schoelkopf, M. H. Devoret, L. I. Glazman, Relaxation and frequency shifts induced by quasiparticles in superconducting qubits. *Phys. Rev. B* **84**, 064517 (2011).

57. A. Somoroff, Q. Ficheux, R. A. Mencia, H. Xiong, R. Kuzmin, V. E. Manucharyan, Millisecond coherence in a superconducting qubit. *Phys. Rev. Lett.* **130**, 267001 (2023).

58. F. Henriques, F. Valenti, T. Charpentier, M. Lagoin, C. Gouriou, M. Martínez, L. Cardani, M. Vignati, L. Grünhaupt, D. Gusenkova, J. Ferrero, S. T. Skacel, W. Wernsdorfer, A. V. Ustinov, G. Catelani, O. Sander, I. M. Pop, Phonon traps reduce the quasiparticle density in superconducting circuits. *Appl. Phys. Lett.* **115**, 212601 (2019).

59. R. T. Gordon, C. E. Murray, C. Kurter, M. Sandberg, S. A. Hall, K. Balakrishnan, R. Shelby, B. Wacaser, A. A. Stabile, J. W. Sleight, M. Brink, M. B. Rothwell, K. P. Rodbell, O. Dial, M. Steffen, Environmental radiation impact on lifetimes and quasiparticle tunneling rates of fixed-frequency transmon qubits. *Appl. Phys. Lett.* **120**, 074002 (2022).

60. X. You, J. A. Sauls, J. Koch, Circuit quantization in the presence of time-dependent external flux. *Phys. Rev. B* **99**, 174512 (2019).

61. J. Braumüller, L. Ding, A. P. Vepsäläinen, Y. Sung, M. Kjaergaard, T. Menke, R. Winik, D. Kim, B. M. Niedzielski, A. Melville, J. L. Yoder, C. F. Hirjibehedin, T. P. Orlando, S. Gustavsson, W. D. Oliver, Characterizing and optimizing qubit coherence based on squid geometry. *Phys. Rev. Appl.* **13**, 054079 (2020).

62. L. B. Nguyen, G. Koolstra, Y. Kim, A. Morvan, T. Chistolini, S. Singh, K. N. Nesterov, C. Jünger, L. Chen, Z. Pedramrazi, B. K. Mitchell, J. M. Kreikebaum, S. Puri, D. I. Santiago, I. Siddiqi, Blueprint for a high-performance fluxonium quantum processor. *PRX Quantum* **3**, 037001 (2022).

63. C. Wang, X. Li, H. Xu, Z. Li, J. Wang, Z. Yang, Z. Mi, X. Liang, T. Su, C. Yang, G. Wang, W. Wang, Y. Li, M. Chen, C. Li, K. Linghu, J. Han, Y. Zhang, Y. Feng, Y. Song, T. Ma, J. Zhang, R. Wang, P. Zhao, W. Liu, G. Xue, Y. Jin, H. Yu, Towards practical quantum computers: Transmon qubit with a lifetime approaching 0.5 milliseconds. *npj Quantum Inf.* **8**, 3 (2022).

64. T. Takahashi, N. Kouma, Y. Doi, S. Sato, S. Tamate, Y. Nakamura, Uniformity improvement of Josephson-junction resistance by considering sidewall deposition during shadow evaporation for large-scale integration of qubits. *Jpn. J. Appl. Phys.* **62**, SC1002 (2022).
